# Restrained Dendritic Growth of Adult-Born Granule Cells Innervated by Transplanted Fetal GABAergic Interneurons in Mice with Temporal Lobe Epilepsy

**DOI:** 10.1523/ENEURO.0110-18.2019

**Published:** 2019-04-09

**Authors:** Jyoti Gupta, Mark Bromwich, Jake Radell, Muhammad N. Arshad, Selena Gonzalez, Bryan W. Luikart, Gloster B. Aaron, Janice R. Naegele

**Affiliations:** 1 Department of Biology, Program in Neuroscience and Behavior, Hall-Atwater Laboratory, Wesleyan University, Middletown, CT 06459-0170; 2 Department of Molecular and Systems Biology, Geisel School of Medicine at Dartmouth, Lebanon, NH 03755-1404

**Keywords:** dendrite, dentate gyrus, GABAergic, hippocampus, neurogenesis, transplant

## Abstract

The dentate gyrus (DG) is a region of the adult rodent brain that undergoes continuous neurogenesis. Seizures and loss or dysfunction of GABAergic synapses onto adult-born dentate granule cells (GCs) alter their dendritic growth and migration, resulting in dysmorphic and hyperexcitable GCs. Additionally, transplants of fetal GABAergic interneurons in the DG of mice with temporal lobe epilepsy (TLE) result in seizure suppression, but it is unknown whether increasing interneurons with these transplants restores GABAergic innervation to adult-born GCs. Here, we address this question by birth-dating GCs with retrovirus at different times up to 12 weeks after pilocarpine-induced TLE in adult mice. Channelrhodopsin 2 (ChR2)-enhanced yellow fluorescent protein (EYFP)-expressing medial-ganglionic eminence (MGE)-derived GABAergic interneurons from embryonic day (E)13.5 mouse embryos were transplanted into the DG of the TLE mice and GCs with transplant-derived inhibitory post-synaptic currents (IPSCs) were identified by patch-clamp electrophysiology and optogenetic interrogation. Putative synaptic sites between GCs and GABAergic transplants were also confirmed by intracellular biocytin staining, immunohistochemistry, and confocal imaging. 3D reconstructions of dendritic arbors and quantitative morphometric analyses were carried out in >150 adult-born GCs. GABAergic inputs from transplanted interneurons correlated with markedly shorter GC dendrites, compared to GCs that were not innervated by the transplants. Moreover, these effects were confined to distal dendritic branches and a short time window of six to eight weeks. The effects were independent of seizures as they were also observed in naïve mice with MGE transplants. These findings are consistent with the hypothesis that increased inhibitory currents over a smaller dendritic arbor in adult-born GCs may reduce their excitability and lead to seizure suppression.

## Visual Abstract

**Figure:**
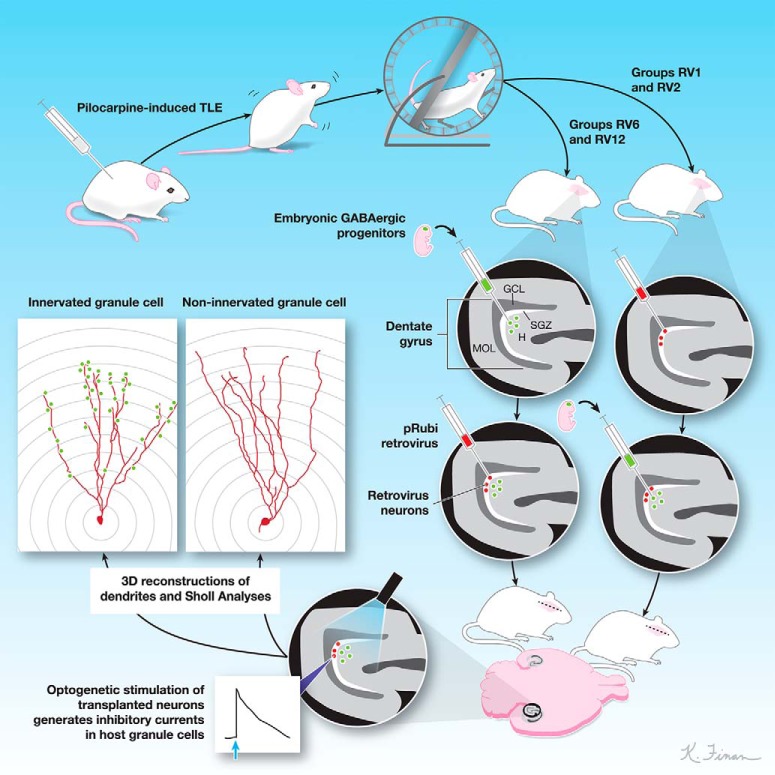

## Significance Statement

Transplants of medial-ganglionic eminence (MGE) GABAergic progenitors into the hippocampus of adult mice with pilocarpine-induced temporal lobe epilepsy (TLE) have been shown to increase inhibitory synaptic currents in granule cells (GCs) in the dentate gyrus (DG) and suppress seizures. Here, we investigated whether the increased transplant-derived inhibition resulted in structural changes to the dendritic arbors of adult-born GCs that might be responsible for reduced excitability. Our results show that transplant-innervated adult-born GCs form significantly shorter dendrites compared to non-innervated GCs. These changes were restricted to distal dendrites in GCs generated within two months after transplantation. These findings suggest a structural mechanism for seizure suppression whereby increased GABAergic innervation from transplanted MGE progenitors may restrict the growth of dendritic arbors in adult-born hippocampal GCs.

## Introduction

Hippocampal neural circuit dysfunction is thought to contribute to epileptogenesis and the development of spontaneous seizures in temporal lobe epilepsy (TLE; Goldberg and Coulter, 2013; Alexander et al., 2016). Mounting evidence suggests that disruptions to the normal pattern of adult neurogenesis in the dentate gyrus (DG) promote the development of hyperexcitability (Parent et al., 1997; Jessberger et al., 2005, 2007). Prolonged seizures, defined as status epilepticus (SE), alter granule cell (GC) neurogenesis in the DG of the hippocampus (Parent et al., 1997; Hattiangady et al., 2004; Jessberger et al., 2005; Hattiangady and Shetty, 2010). Following SE, many adult-born GCs migrate to ectopic locations in the hilus or molecular layer of the DG (Scharfman et al., 2000, 2003b; Scharfman, 2005; Parent et al., 2006) and form basal dendrites that establish abnormal and hyperexcitable neural circuits (Thind et al., 2008; Murphy et al., 2011). Studies have shown that the number of hilar ectopic adult-born GCs positively correlates with the seizure burden, and experimentally ablating GCs born after SE reduces the seizure burden in TLE (Hester and Danzer, 2013; Cho et al., 2015; Hosford et al., 2016, 2017). Paradoxically, although ablating adult-born GCs in rodents with SE reduced the incidence of seizures, it increased the duration of residual seizures, possibly due to eliminating both normotopic and ectopic adult-born GCs (Hosford et al., 2016; Yu and Krook-Magnuson, 2017).

The patterns of excitatory and inhibitory connections to both the adult-born and developmentally-born GCs are also altered in TLE (Du et al., 2017). Several factors contribute to this phenomenon, including mossy fiber sprouting (Okazaki et al., 1995; Buckmaster et al., 2002; Scharfman et al., 2003a; Buckmaster, 2012), dendritic hypertrophy (Patel et al., 2004; Arisi and Garcia-Cairasco, 2007; Walter et al., 2007; Nishimura et al., 2008), and dendritic spine loss (Jiang et al., 1998). Prolonged SE can also trigger excitotoxic cell death of hippocampal GABAergic interneurons and lead to compensatory sprouting of surviving interneurons (Zhang et al., 2009; Long et al., 2011; Peng et al., 2013; Buckmaster et al., 2017). Exactly how each of these changes contributes to hyperexcitability is not fully understood (Beck et al., 1996; Bausch and McNamara, 2000; Cossart et al., 2001; Althaus et al., 2015). However, these and other experimental findings support the hypothesis that SE triggers structural and functional modifications to neural circuits in the DG that promote hyperexcitability and the development of recurrent spontaneous seizures (SRS) in TLE. Considerable research is now focusing on understanding the cellular and molecular mechanisms for these structural and functional changes in TLE (Pun et al., 2012; LaSarge et al., 2015, 2016; Nishimura et al., 2017; Santos et al., 2017). Converging evidence supports the hypothesis that abnormal cellular growth mechanisms and altered gene expression lead to progressive changes that are sufficient to cause recurrent seizures and epilepsy (Parent et al., 1997, 2006; Walter et al., 2007).

Prior work established that transplanting fetal rodent GABAergic interneuron progenitors harvested from the medial-ganglionic eminence (MGE) or purified GABAergic interneurons derived from human embryonic stem cells are effective at suppressing seizures in developmental and chemoconvulsant models of epilepsy (Baraban et al., 2009; Zipancic et al., 2010; Hunt et al., 2013; Cunningham et al., 2014; Henderson et al., 2014; Lee et al., 2014; Shetty and Upadhya, 2016). Some evidence suggests that MGE transplants also reduce mossy fiber sprouting (Henderson et al., 2014; but see Hunt et al., 2013). After transplantation into the hippocampus of mice with TLE, fetal mouse MGE progenitors were linked to increased spontaneous IPSCs in hippocampal GCs (Henderson et al., 2014) and increased phasic and tonic inhibition in other populations of host brain neurons (Baraban et al., 2009; Hsieh and Baraban, 2017) via α4 subunit containing GABA_A_ receptors (Jaiswal et al., 2015). The notion that transplanted neurons integrate into host brain circuits and provide synaptic inhibition is further supported by optogenetic experiments showing that stimulation of transplanted channelrhodopsin 2 (ChR2)-expressing GABAergic neurons induced strong IPSCs in hippocampal neurons (Henderson et al., 2014; Hsieh and Baraban, 2017). Other mechanisms may also be involved, as MGE progenitor grafts were shown to induce critical period plasticity and rewiring in the spinal cord and cerebral cortex (Park et al., 2002; Lee et al., 2007; Southwell et al., 2010; Chohan and Moore, 2016; Spatazza et al., 2017).

As a further step toward defining the cellular mechanisms responsible for fetal GABAergic interneuron-mediated effects in TLE, we examined whether transplantation of mouse MGE progenitors into the DG of naïve or TLE mice altered the morphologic development of adult-born GCs. We found that compared to adult-born GCs that were not innervated by the transplanted cells, the adult-born GCs with fetal transplant innervation exhibited significantly smaller dendritic arbors and shorter distal dendrites. These changes to the dendritic arbors were chiefly within the outer molecular layer of the DG, where adult-born GCs normally receive excitatory inputs from the lateral entorhinal cortex (Woods et al., 2018). The overall growth of dendritic arbors was also reduced in adult-born GCs in naïve, non-epileptic mice that received innervation from transplanted GABAergic interneurons, suggesting a general mechanism whereby inhibitory inputs from transplanted MGE-derived GABAergic interneurons diminish dendritic growth.

## Materials and Methods

Table 1 contains a summary of the experimental groups of mice that were used for electrophysiological recordings and morphology of adult-born GCs. All materials are listed in Table 2 except for common laboratory reagents that were purchased from Sigma.

**Table 1.** Summary table for number of GCs recorded

	Number of mice	Number of recorded RV-labeled GCs	Number of GCs that responded to light stimulation	Number of responsive GCs that were morphologically recovered following electrophysiology and confirmed to be RV labeled
1-week RV	6	23	10	6
2-week RV	5	15	8	2
6-week RV	5	13	8	5
12-week RV	2	4	4	1

**Table 2.** Key resources

Reagent or resource	Source	Identifier
Antibodies		
Anti-GFP (green fluorescent protein; chicken antibodies, IgY fraction)	Aves	GFP-1020
Rabbit mCherry	Invitrogen	PA5-34974
Streptavidin, Alexa Fluor 647 conjugate	Invitrogen	S32357
Alexa Fluor 488 goat anti-chicken (H + L)	Life Technologies	A11039
Alexa Fluor 568 goat anti-rabbit IgG (H + L)	Life Technologies	P36971
Plasmids		
pRubi	Luikart Lab (Dartmouth)	
redRubi	Luikart Lab (Dartmouth)	
Chemicals, media		
ProLong diamond antifade mountant with DAPI	Invitrogen	P36971
Iscove's modification of DMEM	Corning	10-016-CV
L-glutamine, 100×, liquid	Corning	25-005-CI
MEM nonessential amino acids	Corning	25-025-CI
Penicillin-streptomycin solution, 100×	Corning	30-002-CI
PEG 6000, molecular biology grade	Millipore Sigma	528877
L-15 medium (Leibovitz) with L-glutamine	Sigma-Aldrich	SLBR4210V
Defined trypsin inhibitor (1×)	Gibco	R-007-100
2.5% trypsin (10×)	Gibco	15090-046
Mouse EGF	Cell Signaling	5331SF
Fibroblast growth factor-basic human	Cell Signaling	F0291
B-27 supplement	Gibco	17504-044
Caspase inhibitor Z-VAD-FMK 20 mM	Promega	G7231
Commercial kits		
NucleoBond Xtra Maxi DNA, RNA and protein purification kit	Macherey-Nagel	740414.10
Experimental models: cell lines		
293 GP	Luikart Lab (Dartmouth)	
293 R	Luikart Lab (Dartmouth)	
Experimental models: mice		
C57BL/NHsd	Envigo	
B6.Cg-Tg(Slc32a1-COP4*H134R/EYFP)8Gfng/J (VGAT-ChR2-EYFP line 8)	The Jackson Laboratory	014548
Software IMARIS	Bitplane	

### Animals

Animal protocols were approved by the Institutional Animal Care and Use Committee (IACUC). As differences in estrous cycle in female mice were reported to affect seizure susceptibility, we performed all studies in male C57BL/6NHsd adult mice (Envigo) to reduce animal use (Müller et al., 2009). The mice were purchased at four to six weeks of age and maintained singly in self-ventilating cage racks in the animal facility. They were placed on a 12/12 h light/dark cycle and provided with food and water *ad libitum*. The mice were handled daily for one to two weeks before seizures were induced. MGE-derived GABAergic progenitors for transplantation were obtained from timed-pregnant C57BL/6NHsd female mice (Envigo) bred to adult male transgenic mice expressing ChR2-enhanced yellow fluorescent protein (EYFP) under the control of the vesicular GABA promoter (VGAT; VGAT-ChR2-EYFP line 8 (JAX Stock No. 014548, B6.Cg-Tg (Slc32a1-COP4*H134R/EYFP)8Gfng/J; Zhao et al., 2011).

### Pilocarpine and drug administration

The pilocarpine model was used to replicate TLE in six- to eight-week-old male mice (Envigo, C57BL/6N strain, 18–22 g), as described previously (Henderson et al., 2014). Seizures were induced in male mice, since females in this mouse strain show significant differences in seizure latency, which could increase variability in patterns of recurrent seizure severity and incidence (Müller et al., 2009). On the day of seizure induction, the male mice were injected with methyl-scopolamine (0.5 mg/ml, i.p.; Sigma-Aldrich), followed 30 min later by an injection of pilocarpine hydrochloride (280 mg/kg, i.p.; Sigma-Aldrich). One or two supplemental doses of pilocarpine (30–60 mg/kg, i.p.) were administered 30 min after the initial injection, if the mouse failed to develop seizures that progressed to status epilepticus (SE). Seizures were rated behaviorally, based on a modified Racine scale until the animal reached SE (Shibley and Smith, 2002; Henderson et al., 2014). One hour after continuous SE, seizures were attenuated by injecting midazolam (9.1–11.1 mg/kg, i.p.; Akorn). Ringer’s solution (1 ml, i.p.; Henry Schein) was administered as needed, until recovery. A total of 124 mice were induced by pilocarpine. Of these, 77 developed SE (63%), 47 either died during SE or failed to develop SE and were euthanized (37%). Of the 77 SE mice, 44 underwent stereotaxic surgery to inject retrovirus and MGE-derived GABAergic progenitors in the DG and 41 of these mice were then used for slice electrophysiology and neuronal reconstructions. A total of 17 C57BL/6N male mice (six- to eight-week-old, 18–22 g) did not receive pilocarpine and were used as naïve, non-epileptic controls; 13 underwent stereotaxic surgery to inject retrovirus and MGE-derived GABAergic progenitors in the DG of the hippocampus. These mice were subsequently used for slice electrophysiology and neuronal reconstructions. Three additional SE mice received MGE-derived GABAergic progenitor transplants and were used for immunohistochemical analyses of the transplanted cells.

### Viral production and injection

We labeled adult-born GCs in naïve mice and in mice with pilocarpine-induced TLE by stereotaxic injections of a Moloney murine leukemia virus (MMLV)-based retroviral vector (Luikart et al., 2011, 2012) into the subgranular zone of the DG. In mice with SE, the times of retrovirus (RV) injections were selected to coincide with early stages of seizure-induced upregulation of adult neurogenesis (one or two weeks after induction of SE) or the chronic phase of TLE (six or 12 weeks post-SE).

The MMLV-based retrovirus was produced by transient transfection of GP-293 retroviral packaging cell line with a retrovirus with internal ubiquitin promoter expressing mCherry (redRubi) and vesicular stomatitis virus glycoprotein (VSVg). The supernatant containing the viral particles was purified using PEG6000, as described previously (Luikart et al., 2011, 2012). The retrovirus titer produced in our laboratory was 1–10 × 10^8^ transfection units per milliliter (TU/ml).

Twenty-four hours before stereotaxic surgery, the mice were given overnight access to running wheels (ENV-044, ENV-044-02; Med Associates Inc.) to increase neurogenesis (Holmes et al., 2004). Thirty minutes before stereotaxic injections of virus, the mice were injected with Meloxicam (0.03 ml, s.c.; Boehringer Ingelheim). Anesthesia was induced by isoflurane inhalation (Isothesia, Henry Schein) and lidocaine hydrochloride was applied to the skin overlying the midline of the skull for topical anesthesia (2% topical; Hi·Tech Pharmacal). Each mouse received eight stereotaxic injections of retrovirus (0.5 µl per site); the injections were made bilaterally into four sites in each hippocampus at a rate of 0.25 µl/min with a glass syringe (10 µl, removable needle, Hamilton) equipped with a 30-gauge needle (30 GA, 1”, point style 2, 45°, Hamilton), at the following coordinates: AP –1.9 mm, ML ±1.1, DV –2.5 and –2.3; AP –2.5, ML ±2.1, DV –2.2 and –1.8. For two injection sites along one needle track, the first injection was made into the more ventral location and the needle remained in place for 2 min before a second injection was made into the more dorsal location. The needle was left in place for 5 min before being withdrawn and the skin incision was sealed (VetBond tissue adhesive, 3M Corp.). Mice were kept on a heating pad until fully awake and then housed in their home cages.

### Transplantation of MGE-derived GABAergic progenitors

MGE-derived GABAergic progenitors were transplanted two weeks post-SE in mice receiving retrovirus at one, six, or 12 weeks post-SE, except for one group of mice that received retrovirus injections two weeks post-SE; in these mice the GABAergic progenitors were transplanted six weeks post-SE. Donor cells were harvested from the MGE of embryonic day (E)13.5 transgenic mouse embryos derived from adult female breeders (Envigo, C57BL/6NHsd) bred to VGAT-ChR2-EYFP line 8 (JAX Stock No. 014548, B6.Cg-Tg(Slc32a1-COP4*H134R/EYFP)8Gfng/J; Zhao et al., 2011). EYFP-positive embryos were identified with specialized goggles (FHS/F-01 headlamp equipped with FHS/EF-2G2 emission filters, Biological Laboratory Equipment Maintenance and Service Ltd.). The MGE on each side of the embryonic brain was isolated by free-hand dissection in cold HBSS (Sigma) without calcium or magnesium, as described previously (Xu et al., 2004; Henderson et al., 2014; Vogt et al., 2015). The tissue was incubated in 0.125% trypsin (Gibco) in HBSS at 37°C for 12 min, transferred with fire-polished glass pipettes into trypsin inhibitor (1×, Gibco) for 10 min, then mechanically dissociated first with a large bore (10 times) then a small bore, fire-polished glass pipette (10 times). The dissociated cells were centrifuged and suspended in cell transplantation media. Cell counts were performed with a hemocytometer and the cells were then further diluted in cell transplantation media to reach a final concentration of 1 × 10^5^ cells/μl. The transplantation media consisted of L-15 supplemented with fibroblast growth factor (Cell Signaling), epidermal growth factor (Cell Signaling), Caspase Inhibitor (Promega) and B27 (Gibco), as described previously (Henderson et al., 2014). The cell suspension was maintained on ice during determination of cell number and then 1 µl (containing ∼100,000 cells) was slowly injected over the course of 5 min into a single injection site into the hilus of the DG, by means of stereotaxic surgery. Bilateral injections were made into each DG (stereotaxic coordinates AP −2.5 mm, ML ±2.1 mm, DV −2.2 and −1.8 mm). The needle was left at the injection site for 5 min before being withdrawn. The skin incision was closed, and the mice were allowed to recover on a heated pad, then returned to their cages.

### Hippocampal slice electrophysiology

After allowing approximately eight weeks for maturation of adult-born GCs and interneuron transplants, we performed whole-cell patch-clamp recordings of GCs in hippocampal slices, as described previously (Henderson et al., 2014). The mice were anesthetized with a mixture of ketamine hydrochloride (120 mg/kg, i.p.; Ketaset, Zoetis) and xylazine (10 mg/kg, i.p.; Anased, Lloyd Laboratories), before rapidly removing the brain, and transferring it to oxygenated, ice-cold ACSF (high sucrose ACSF; 27.07 mM NaHCO_3_, 1.5 mM NaH_2_PO_4_, 1 mM CaCl_2_, 3 mM MgSO_4_, 2.5 mM KCl, and 222.14 mM sucrose). Brains were mounted onto the chilled stage of a vibratome (Leica VT1000S) in the horizontal plane, bisected in the sagittal plane, and thick slices (350 µm) were cut from ventral to dorsal. The slices were maintained in oxygenated ACSF (37°C; 125 mM NaCl, 1 mM CaCl_2_, 3 mM MgSO_4_, 1.25 mM NaH_2_PO_4_, 25 mM NaHCO_3_, 2.5 mM KCl, 25 mM glucose, 3 mM myo-inositol, 2 mM Na-pyruvate, and 0.4 mM ascorbic acid) and for electrophysiology, slices were individually transferred into a recording chamber containing oxygenated ACSF (125 mM NaCl, 1.5 mM CaCl_2_, 1.0 mM MgSO_4_, 1.25 mM NaH_2_PO_4_, 25 mM NaHCO_3_, 3.5 mM KCl, 25 mM glucose, 3 mM myo-inositol, 2 mM Na pyruvate, and 0.4 mM ascorbic acid). Electrophysiological recordings were obtained from retrovirally (RV)-labeled GCs and unlabeled GCs in voltage-clamp mode at 34°C using a Cesium gluconate intracellular solution (135 mM gluconic acid, 135 mM CsOH, 1 mM EGTA, 8 mM MgCl, 0.1 mM CaCl_2_, 10 mM HEPES, 2 mM Mg-ATP, and 0.3 mM Na-GTP) containing biocytin (11 mM, Sigma), to allow us to recover and stain the electrophysiologically-recorded cells at the end of the experiment. To record IPSCs, the cells were voltage clamped at +10 mV. To examine putative synaptic connections between the transplanted MGE-derived interneurons and GCs, we optogenetically activated transplanted ChR2-expressing interneurons using blue light pulses and recorded the responses in DG GCs, as described previously (Henderson et al., 2014). The blue light stimulus was triggered using a Master 8 stimulator (AMP instruments) and consisted of five pulses of blue light (5-ms duration each) with an interstimulus interval of 200 ms (Henderson et al., 2014).

### Analysis of electrophysiological recordings

The electrophysiological recordings were analyzed offline using IGOR software. The amplitude of each light-induced IPSC was measured. For each GC, the amplitude of induced IPSCs was determined by taking the average of all the induced IPSCs across a trial. We also determined the IPSC latency to the light stimuli by measuring the delay between onset of the light stimulus and IPSC onset in all adult-born GCs that were confirmed to be mCherry^+^ and thus RV labeled. To calculate the relationship between the magnitude of the light-induced currents in GCs and the number of MGE transplant-derived synaptic puncta, we correlated the peak amplitude of light-induced currents to the total number of synaptic appositions counted with the Spots function in IMARIS software, as described below.

### Immunohistochemical staining of thick slices

Following electrophysiological recordings, the slices were fixed overnight in 4% paraformaldehyde (Electron Microscopy Sciences). The slices were then equilibrated in PBS containing 30% sucrose and frozen in tissue freezing medium (General Data).

To confirm RV labeling in electrophysiologically-identified GCs within the vicinity of transplants of MGE-derived interneurons, we immunostained all the slices for mCherry, eYFP and biocytin. The slices were thawed and transferred to blocking buffer (5% normal goat serum containing 0.3% Triton-X) for 1 h at room temperature (RT). Slices were incubated for 48 h at RT in primary antibody solution in blocking buffer containing: chicken anti-GFP (1:1000, Aves), rabbit anti-mCherry (1:1000, Invitrogen), and streptavidin Alexa Fluor 647 (1:500, Invitrogen; omitted when staining slices that did not contain biocytin-filled cells). The slices were then washed for 1 h in PBS (KPBS, 0.02 M) and transferred into secondary antibody solution containing goat anti-rabbit Alexa Fluor 568 (1:1000, Life Technologies) and goat anti-chicken Alexa Fluor 488 (1:1000, Life Technologies) for 24 h, before a final wash for 1 h. Sections were mounted on Superfrost Plus slides in Prolong Gold with DAPI (Invitrogen) and stored at –20°C in the dark.

### Phenotypic characterization of transplanted cells

Three additional SE mice with MGE-derived GABAergic progenitor transplants were perfused with 4% paraformaldehyde in 0.1 M phosphate buffer (pH 7.4), and the brains were equilibrated in ascending sucrose solutions (10%, 20%, and 30% sucrose in 0.1 M phosphate buffer, pH 7.4), then frozen in tissue freezing media (General Data). Forty-micrometer-thick cryostat sections were collected in 0.1 M PBS and immunofluorescent staining was performed to detect transplanted GABAergic interneurons expressing ChR2-EYFP (chicken anti-GFP, GFP 1020, Aves), in combination with one of the following neurochemical markers: parvalbumin (PV; rabbit anti-PV, 1:100, PV-27, Swant), somatostatin (SOM; rat anti-SOM, 1:100, mab354 Millipore), calretinin (CR; rabbit anti-CR, 1:100, ab704, Abcam), or neuronal nitric oxide synthase (nNOS; rabbit anti-nNOS, 1:1000, AB5380, Millipore). Incubations were performed on free-floating sections at 4°C for 48 h with agitation. Sections were washed in PBS and secondary antibodies were applied. Nuclei were labeled with NeuroTrace Nissl 647 (1:300, Life Technologies), and sections were mounted onto glass slides in Prolong with DAPI (Life Technologies). We quantified the neurochemical phenotypes of all single and double-labeled cells contained in the hippocampus in a total of 20–24 sections/mouse. For each mouse, six sections spaced 400 μm apart were immunostained for EYFP and one of the four neurochemical markers. Using optical slices obtained from confocal microscopic images of the hippocampal sections, we quantified the total number of individual, transplanted EYFP^+^ interneurons in each hippocampus and the number that co-expressed an additional neurochemical marker.

### Confocal imaging

For imaging biocytin-filled GCs and the neurochemical phenotypes of transplanted cells, we collected optical slices through the entire thickness of 350-μm-thick fixed and stained hippocampal slices that had been used for electrophysiological studies. Confocal microscopy (Zeiss LSM 510 or a Leica SP8) was performed at 0.5 μm steps using a 25× objective (Zeiss Plan-Neofluar 25×, N.A. 0.8, water immersion objective) and a 63× objective (Zeiss C-Apochromat 63× objective, N.A. 1.2, water immersion). For Sholl analyses, RV-labeled GCs were imaged at lower magnifications to record their positions and capture the entire dendritic arbors (Zeiss Plan-Apochromat 10×, N.A. 0.45 and Zeiss Plan-Neofluar 25×, N.A. 0.8, water immersion objectives; z-stacks with a step size of 1 µm and frame size, 1024 × 1024 pixels). For detection and quantification of putative synaptic contacts formed by the transplanted GABAergic interneurons onto RV-labeled GCs, we imaged each RV-labeled neuron at high magnification (C-Apochromat 63× objective, N.A. 1.2, water immersion). These high-resolution images were obtained as z-stacks with a step size of 0.3 µm and resolution of 2048 × 2048 pixels. All confocal images were stored on local hard drive (Backup Plus Desktop Drive, Seagate Technology) and archived in long-term cloud storage (RStore).

### 3D reconstructions and dendritic arbor morphometric analyses

To analyze dendritic morphology in RV-labeled adult-born GCs, confocal z-stacks of cells were imported into IMARIS neuron reconstruction software (8.4.0, Bitplane). The dendrites were traced using the filament tracer tool. Morphometric analyses of the reconstructed dendritic arbors were performed to quantify the following cell properties: total dendritic length, number of different order branches, length of different branches, and number of branch points. For dendritic branch lengths and total dendritic lengths, the distance from the center of the soma to the beginning of the primary dendrite was excluded. To determine dendritic complexity, 3D Sholl analyses were performed from the center of GC somas in IMARIS, identifying dendritic branch intersections at 10-µm intervals.

Because electron microscopy has not been used to confirm that the axonal swellings formed by the transplanted neurons onto the GCs in the host brains are bona fide inhibitory GABAergic synapses, we refer to these morphologic swellings as “putative synapses” throughout the article. To quantify putative transplant-derived synapses onto RV-labeled GCs, the Spots function in IMARIS was used to identify green fluorescent boutons within 0.5 μm of the cell that had a diameter of ∼0.75 μm or larger. Each putative synapse was checked manually to eliminate false signals. It seems probable that these putative synapses are sites of neurotransmitter release, as it was previously demonstrated by optogenetic studies that light-induced depolarization of the transplanted cells induced strong IPSCs in nearby GCs with short latencies (Henderson et al., 2014). Additionally, ∼60% of these axonal swellings were previously found to be closely associated with postsynaptic clusters of gephyrin, an inhibitory synapse-specific postsynaptic density protein (Henderson et al., 2014).

We further classified the RV-labeled adult-born GCs based on patterns of innervation; the adult-born GCs that had fewer than 15 transplant-derived boutons on the entire cell were classified as “non-innervated” by the transplanted cells whereas GCs with >15 transplant-derived putative synapses were classified as “innervated.” The Sholl analyses were performed on adult-born GCs that were RV-labeled after SE as follows: RV1 week: 42 GCs (29 non-innervated and 13 innervated), RV2 weeks: 27 GCs (16 non-innervated and 11 innervated), RV6 weeks: 30 GCs (20 non-innervated and 10 innervated), and 12 weeks: 27 GCs (10 non-innervated and 17 innervated).

### Statistical analyses

All statistical analyses were performed in SAS, version 9.4 (SAS Institute). Sholl intersection data were analyzed using a mixed-effects model with an auto-regressive variance structure and multi-level nesting along with the use of MULTTEST procedure to correct for false discovery rates, due to multiple t-tests (Wilson et al., 2017). Analyses of the length and numbers of different order branches were also performed using a mixed model. Comparisons of the means of the total dendritic lengths across different groups were done with ANOVA. Data are presented as the mean ± SEM.

## Results

### What is the spatiotemporal pattern of adult-neurogenesis in the hippocampus at different times after SE?

**Figure 1.**
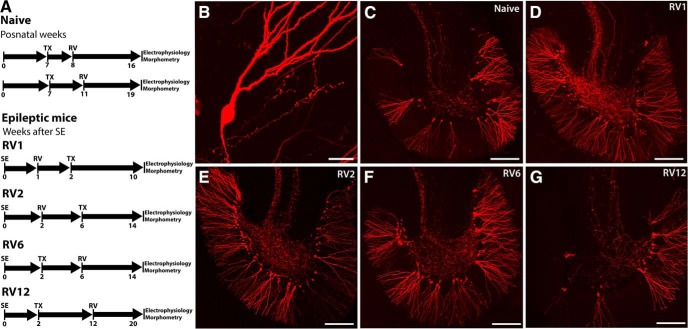
Experimental timeline for RV labeling of adult-born GCs in naïve and SE mice. ***A***, Different groups of mice studied and the experimental timelines for each group including: timing of retroviral injections (RV), day of transplantation of E13.5 MGE progenitors (TX), day of optogenetic and patch-clamp electrophysiological experiments, and 3D neuronal reconstructions and Sholl analyses. ***B***, RV expression (mCherry, red) in a dentate GC showing labeling of dendritic arbor and axon. ***C***, Patterns of RV-labeled GCs in an adult naïve mouse. ***D–G***, Patterns of RV labeling in adult-born GCs in TLE mice following retroviral injections at one, two, six, or 12 weeks post-SE. Scale bars: 20 µm (***B***) and 200 µm (***C–G***).

Given that adult-born GCs display morphologic and functional abnormalities and are believed to contribute to hyperexcitability in the pilocarpine model, we first characterized adult-born GCs generated in naïve and epileptic mice at different times after SE by RV labeling and confocal microscopy. To this end, we made RV injections into the subgranular zone of the DG in naïve and epileptic mice at one, two, six, or 12 weeks after inducing SE and allowed one to two months for the adult-born neurons to reach maturity before analyzing patterns of neurogenesis (Fig. 1*A*). Retroviral expression of mCherry completely filled the soma, dendrites and frequently the axons of adult born GCs (Fig. 1*B*). In naïve mice, we observed strong labeling of GCs throughout the GC layer (GCL) of the DG (Fig. 1*C*). In epileptic mice, robust labeling of adult-born GCs was observed in the GCL and occasionally in the hilus or subgranular zones of the DG, following retroviral injections at one, two, or six weeks post-SE (Fig. 1*D–F*). In agreement with prior studies, chronically epileptic mice injected with retrovirus 12 weeks post-SE exhibited sparser labeling with more frequent clumps of adult-born GCs (Fig. 1*G*).

### Do adult-born GCs receive functional inhibition from developing MGE transplants?

Given that MGE-derived GABAergic interneuron progenitors take approximately four weeks to mature after transplantation (Southwell et al., 2010), we allowed approximately eight weeks for both the transplanted cells and the RV-labeled GCs to mature. The degree of inhibitory synapse formation from the transplants onto adult-born GCs was assessed first by combining optogenetic stimulation with patch-clamp recordings in acute hippocampal slices and second, by immunostaining the fixed slices and performing high-resolution confocal imaging.

**Figure 2.**
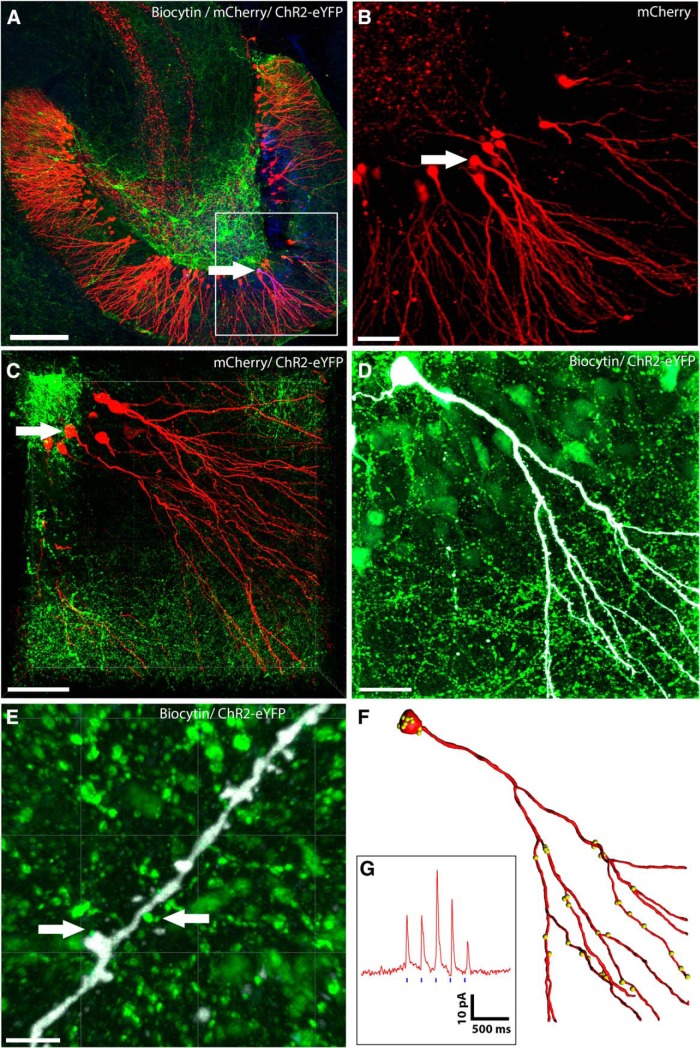
Functional inhibition from transplanted ChR2-expressing MGE-derived GABAergic interneurons onto a mature adult-born GC labeled with RV one week post-SE. Transplanted interneurons were optogenetically stimulated while performing patch-clamp recordings from adult-born, RV-labeled GCs in hippocampal slices. ***A***, Low-magnification image of the DG in an SE mouse that received injections of RV at one week post-SE and a transplant at two weeks post-SE. Boxed area shows RV-labeled GCs (red), and white arrow indicates the RV-labeled GC that was further characterized by patch-clamp electrophysiology, optogenetic stimulation, and biocytin staining (blue). Various interneuron subtypes comprising the transplant are shown in Extended Data Figure 2-1. ***B***, Higher magnification view of the boxed region in ***A***; the electrophysiologically-characterized and biocytin-filled GC is indicated by a white arrow in the group of adult-born GCs (red). ***C***, Magnified view of the same group of RV-labeled adult-born GCs (red) showing the presence of dense plexus of MGE-derived ChR2-EYFP-expressing GABAergic axons (green). Note that due to photobleaching, the density of axons appears to be reduced around the cell bodies. ***D***, Same biocytin-filled GC (white) surrounded by transplanted MGE-derived ChR2-EYFP-expressing GABAergic interneurons and their axonal arbors (green). ***E***, High-magnification single optical slice showing dendritic segment of the biocytin-filled GC (white) and transplant-derived putative synaptic boutons (green). Arrows indicate sites of putative synaptic contacts by the transplanted interneurons onto this GC. ***F***, A partial reconstruction of the same biocytin-filled GC (red). The yellow dots indicate the locations of putative inhibitory synaptic contacts from the transplants. ***G***, Electrophysiological recording of this GC showing optogenetically-induced IPSCs, following blue-light stimulation of the ChR2-expressing transplanted interneurons (vertical blue bars indicate blue light pulses of 5-ms duration and 200-ms interpulse interval). Scale bars: 200 µm (***A***), 50 µm (***B***, ***C***), 20 µm (***D***), and 2 µm (***E***).

We first measured the extent of light induced inhibitory currents in GCs, by recording from RV-labeled, adult-born GCs by whole-cell patch-clamp recordings, while optogenetically activating slices containing ChR2-expressing interneuron transplants (Fig. 1*A*, timeline). A representative slice showing transplanted GABAergic interneurons in the hilus and RV-labeled adult-born GCs in the GCL, is shown in Figure 2*A*. An example of a RV-labeled GC born one week post-SE that was filled with biocytin and further studied by electrophysiology is shown (Fig. 2*B–D*, insets, magnified views,). This neuron received putative transplant-derived synapses onto the dendritic shafts and spines (Fig. 2*E*, arrows; Chiu et al., 2013). Optogenetic stimulation of transplant-derived interneurons induced strong IPSCs in this adult-born GC (Fig. 2*G*). A complete reconstruction of the dendritic arbor revealed 74 transplant-derived, putative synaptic contacts (Fig. 2*F*). We conducted additional electrophysiological studies in 23 hippocampal slices from six mice with RV labeling at one week post-SE and transplants at two weeks post-SE. We successfully recorded from 23 mature GCs in these slices and 10 showed IPSCs in response to optogenetic stimulation. Six of the GCs in this subset were also RV labeled. Dendritic branching patterns of all six GCs were reconstructed in IMARIS and we found an average of ∼180 putative synapses from the transplanted interneurons onto these GCs and a mean light-induced IPSC amplitude of 63.79 ± 28.42 pA. These results, summarized in Table 1, establish that GCs born one week post-SE are heavily innervated by MGE transplants made a week later and that activating the putative synaptic inputs onto these cells induces powerful postsynaptic inhibition.

10.1523/ENEURO.0110-18.2019.f2-1 Extended Data Figure 2-1 Transplanted MGE-derived ChR2-EYFP-expressing GABAergic interneuron progenitors differentiate into multiple subtypes of GABAergic interneurons. ***A–D***, Examples of transplanted interneurons that co-expressed EYFP and PV (*n* = 3 mice; 207 cells) in ML and GCL of the DG. A magnified view of a representative cell is shown in ***D***. ***E–H***, MGE-derived GABAergic interneurons expressing SOM (*n* = 3 mice; 232 cells) were localized primarily near the injection site in the DG. A magnified view of a representative SOM^+^ cell is shown in ***H***. ***I–L***, Examples of transplanted nNos^+^ MGE-derived interneurons (*n* = 3 mice; 73 cells). These cells were located in the ML and hilus of the DG; a magnified image of a representative nNos^+^ cell is shown in ***L***. ***M–O***, Some MGE-derived cells in the grafts expressed CR (*n* = 3 mice; 52 cells) and were localized in the ML and hilus. The strong band of CR^+^ staining in ***N*** is from CR^+^ axons. ***P***, A magnified view of the CR^+^ cell shown in ***M–O***. ***Q***, Quantification of the proportions of each cell type were as follows: 37.3% PV^+^, 33.8%, SOM^+^, 12.4% nNos^+^, and 7.9% CR^+^. ***R–T***, Example of an MGE-derived transplanted interneuron that was characterized by patch-clamp electrophysiology and stained with biocytin. ***R***, Boxed region shows a low-power view of this large interneuron within a transplant. ***S***, The inset from ***R*** shows a higher-magnification image of the transplanted interneuron. ***T***, An IMARIS-based reconstruction of the transplanted interneuron from ***R–T***. Arrows, VGAT-ChR2-EYFP interneurons co-expressing indicated neurochemical markers. Scale bars: 50 µm (***A–C***, ***E–G***, ***I–K***, ***M–O***, ***S***), 20 µm (***D***, ***H***, ***L***, ***P***), and 200 µm (***R***). ML, molecular layer. Download Figure 2-1, TIF file.

### What functional subtypes of interneurons are present in the transplants?

To further characterize the transplanted GABAergic interneurons that could be responsible for driving the observed IPSCs in adult-born GCs, we evaluated the neurochemical subtypes of interneurons within the transplants. We selected molecular markers with little to no overlap in different classes of MGE-derived GABAergic interneurons and show typical immune-staining patterns in Extended Data Figure 2-1. Approximately 37% of the grafted neurons co-expressed VGAT and PV (207 PV^+^/554 EYFP^+^ cells) and 34% co-expressed VGAT and SOM (232 SOM^+^/685 EYFP^+^ cells). A smaller percentage of ∼12% co-expressed VGAT and nNos (73 nNos^+^/588 EYFP^+^ cells), and ∼8% co-expressed CR (52 CR^+^/651 EYFP^+^ cells). While the immunohistochemical data show that ∼71% of the transplanted GABAergic interneurons were either PV- or SOM-positive, any or all of these interneuron subtypes were associated in close proximity with the adult-born GCs.

### Do MGE transplants innervate GCs born at asynchronous stages of development?

**Figure 3.**
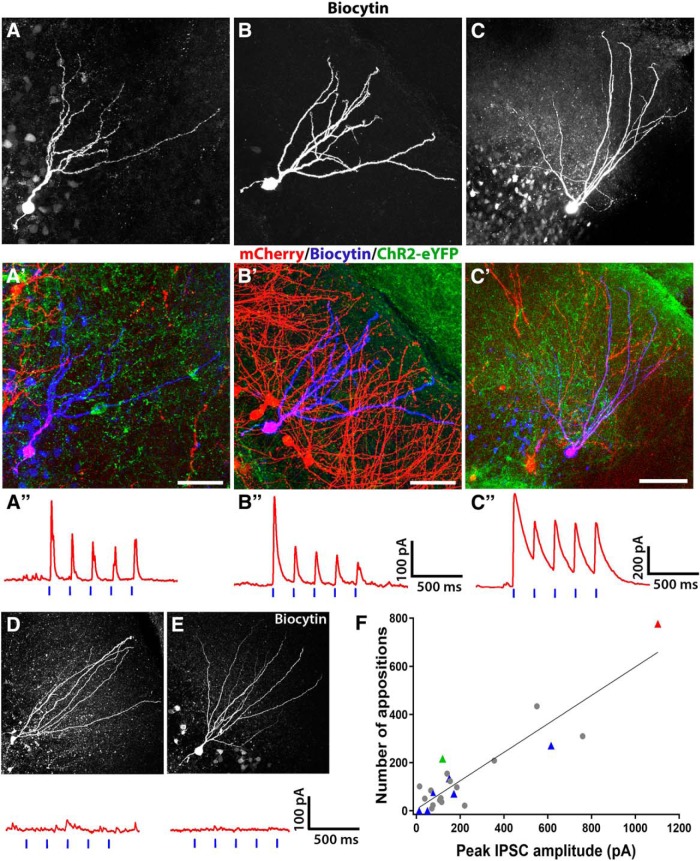
Amplitudes of optogenetically-activated IPSCs in GCs strongly correlate with number of synaptic inputs from transplanted GABAergic interneurons. ***A–C***, Optogenetically-induced synaptic currents were measured in adult-born GCs generated two, six, or 12 weeks post-SE, by whole-cell patch clamping and subsequently filled with biocytin (white). ***A’–C’*,** Merged images of filled GCs showing mCherry expression (red), biocytin-fill (blue), and transplant arborization (green) in these slices. ***A’’–C’’***, Synaptic innervation of adult-born GCs shown in ***A–C*** demonstrated by optogenetic activation of transplanted interneurons. ***D***, ***E***, GCs located outside of the region innervated by the transplanted GABAergic interneurons did not show optogenetically-induced currents. ***F***, Summary graph from all recorded GCs (both RV-labeled and non-labeled) showing strong correlation between the density of transplant-derived boutons and the peak amplitude of light-induced inhibitory currents. Adult-born GCs with confirmed RV labeling are indicated by colored triangle-shaped symbols. The blue triangles are GCs labeled one week post-SE; the green triangles are GCs labeled two weeks post-SE; the red triangles are GCs labeled 12 weeks post-SE. The gray circles are GCs with unknown birthdates.

To determine whether transplanted GABAergic interneurons can innervate adult-born GCs at different stages of maturity, we made retroviral injections at two weeks post-SE and transplants at six weeks post-SE, and then recorded from GCs in these mice four to eight weeks later. In 8/15 cells with strong IPSCs in response to optogenetic stimulation (Fig. 3*A–A’’*), *post hoc* staining confirmed RV labeling in 2/8 (mean IPSC amplitudes: 83.8 and 127.4 pA, respectively). These results suggest that optogenetic stimulation of the transplanted cells induced strong inhibition in many of the adult-born GCs (Fig. 3*A–C*).

### Do later-born GCs receive functional inhibition after MGE transplants have matured?

An additional 13 GCs were recorded from mice with retroviral injections at six weeks post-SE and transplants at two weeks post-SE (Fig. 3*B–B”*), eight were confirmed to have RV labeling, and 5/8 showed light-induced IPSCs (mean IPSC amplitude 175.8 ± 51.4 pA). To test whether GCs born long after transplants were made would show strong transplant-derived synaptic inhibition, we characterized adult-born GCs in mice that received transplants at two weeks post-SE and retroviral injections at 12 weeks post-SE. Remarkably, we found light-induced inhibitory currents in four of these RV-labeled GCs (185.17 ± 89.27 pA; Fig. 3*C–C*” Movie 1). We were further able to confirm RV labeling *post hoc* in one of these cells. As a whole, these findings show that transplanted GABAergic interneurons are capable of providing functional inhibition onto GCs born several months after development of TLE. In the adult-born GCs that did not show light induced currents (Fig. 3*D*,*E*), confocal microscopy showed that the cells generally had <15 transplant-derived synaptic boutons onto their somas or dendrites. In total, we recorded from 55 GCs in mice with retroviral birth-dating at one, two, six, or 12 weeks post-SE. Of the 55 GCs with strong light-induced inhibitory currents, we confirmed retroviral mCherry expression in 14/55 by *post hoc* immunostaining. Twelve of the 14 RV-labeled GCs that were optogenetically responsive showed a delay of 3 ms or less (mean 1.6 ms, range 1–3 ms) between the beginning of blue light stimulus and IPSC onset, indicating a monosynaptic origin of the optogenetically-induced inhibitory currents onto them. Together, these findings indicate that MGE-derived interneurons continue to innervate and form functional inhibitory connections with adult-born GCs over a relatively long period, in excess of 10 weeks after transplantation.

Movie 1. 3D animation of an adult-born GC (red) that was labeled by an RV injection into the dentate gyrus subgranular zone. This GC was born 12 weeks after SE and 10 weeks after transplantation of MGE-derived GABAergic progenitors. The animation shows sites of putative transplant-derived synaptic boutons terminating across this cell’s soma and dendrites (yellow). Optogenetic stimulation of the transplanted interneurons near this GC resulted in robust, short-latency IPSCs in the cell (Fig. 3 *C*, *C’*, *C”*), confirming input from the transplanted interneurons. Only the soma and proximal dendrites are shown in this animation and the complete reconstruction is shown in the extended data in Fig. 6-5. 10.1523/ENEURO.0110-18.2019.video.1

### What is the relationship between the number of synaptic inputs from the transplants and light-induced IPSCs?

Having established that transplanted GABAergic interneurons induce hyperpolarizing currents in GCs born at different times after the development of TLE and transplantation, we next asked whether the number of putative synaptic inputs correlated with the strength of the postsynaptic inhibition, which would further strengthen the evidence that the MGE cells formed functional inhibitory synapses onto adult-born GCs. To address this question, we completed high-resolution reconstructions of 24 GCs from 13 mice that were optogenetically stimulated during electrophysiological recordings. As shown in Figure 3, the number of morphologically-identified putative synaptic boutons onto individual GCs ranged from 14 to 1103 putative synapses/cell. Moreover, the number of these inputs was highly correlated with the peak amplitudes of optogenetically-induced IPSCs onto each cell (*R*^2^ = 0.838; Fig. 3*F*). Remarkably, the largest number of transplant-derived synaptic boutons (1103) was onto a GC born 10 weeks after the GABAergic interneurons were transplanted in a TLE mouse (12 weeks post-SE; Fig. 3*C*). These findings in chronically epileptic mice indicate that the strength (amplitude) of the light-induced inhibitory synaptic currents in adult-born GCs is strongly related to the density of innervation from transplanted interneurons and that long after transplantation, MGE-derived GABAergic interneurons retain a robust capacity for forming putative inhibitory synapses with new adult-born GCs.

### Does functional innervation of adult-born GCs require epilepsy-induced circuit reorganization in host brain?

**Figure 4.**
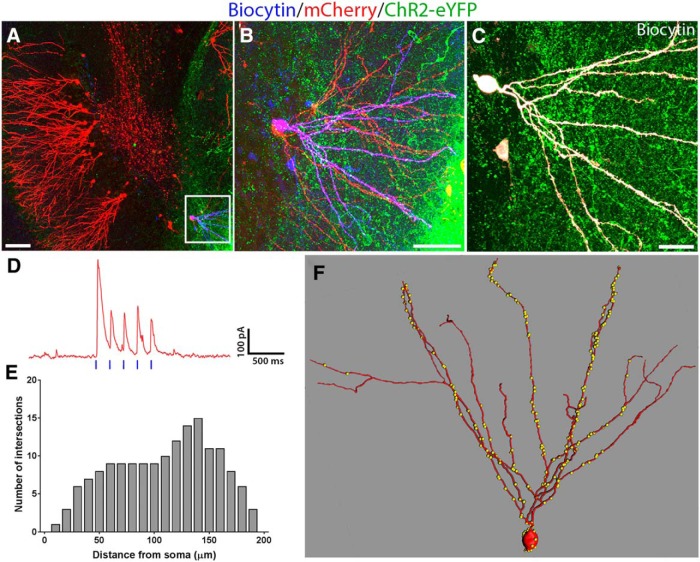
Transplanted GABAergic interneurons innervate adult-born GCs in naïve mice. ***A***, Low-magnification image showing RV-labeled adult-born GCs (red) surrounded by a transplant of ChR2-eYFP-expressing interneurons (green) in the DG of a naïve mouse. Boxed region in ***A***, biocytin-filled (blue) and RV-labeled (red) adult-born GC that was characterized by whole-cell patch clamp electrophysiology. ***B***, Magnified view of the boxed region in ***A***, the same biocytin-filled GC is shown to co-express mCherry. ***C***, Biocytin-filled GC (pseudo-colored white for enhanced visibility) surrounded by neuropil from transplanted MGE-derived GABAergic interneurons (green). ***D***, Electrophysiological recording from this GC showing postsynaptic IPSCs induced by exciting the ChR2-expressing interneurons in this transplant. Vertical blue bars indicated blue light pulses delivered to the slice. ***E***, Sholl analyses of the dendritic arbor of this GC provide quantification of dendritic crossings every 10-µm interval from the cell’s soma. ***F***, IMARIS 3D reconstruction of this GC showing distribution of putative transplant-derived synapses (yellow dots). Scale bars: 100 µm (***A***), 50 µm (***B***), and 20 µm (***C***).

Many studies have shown that pilocarpine-induced SE results in selective loss of SOM^+^ interneurons in the hippocampus and sprouting of subsets of interneurons, as well as other host brain changes (Peng et al., 2013). To determine whether changes in the host brain in TLE are required for the transplanted cells to form synaptic inputs onto adult-born neurons, we studied the innervation of adult-born GCs in naïve, non-epileptic mice by RV labeling adult-born GCs in six- to eight-week-old naïve mice and then a week later, we transplanted fetal GABAergic interneuron progenitors into the DG. After allowing eight weeks for maturation of the RV-labeled GCs and transplanted GABAergic interneurons, we made patch-clamp recordings from the labeled GCs, while activating the slices with blue light. Of 39 GCs recorded in slices from 11 naive mice with transplants, the majority showed robust light-induced responses (24/39 cells, mean IPSC amplitude: 76.15 ± 20.51 pA). One representative example of an RV-labeled, adult-born GC from a naïve mouse is shown in Figure 4*A–C*. This GC showed strong light-induced IPSCs (Fig. 4*D*) and had extensive transplant-derived putative synapses onto its soma and dendrites (Fig. 4*F*). Based on our Sholl analyses, this GC had a typical branching profile (Fig. 4*E*). In just under half (42%) of the 31/39 adult-born GCs with confirmed RV labeling in naïve mice, we were able to demonstrate postsynaptic inhibition by optogenetically stimulating the transplants (13/31 RV-labeled GCs). The remaining 18 RV-labeled GCs were non-innervated and served as an internal control group (also see Fig. 6). Taken together, these results demonstrate that MGE-derived GABAergic progenitors transplanted into the adult DG robustly innervate adult-born GCs in both epileptic and naïve, non-epileptic adult mice.

### Does innervation by MGE transplants alter the dendritic morphology of adult-born GCs?

**Figure 5.**
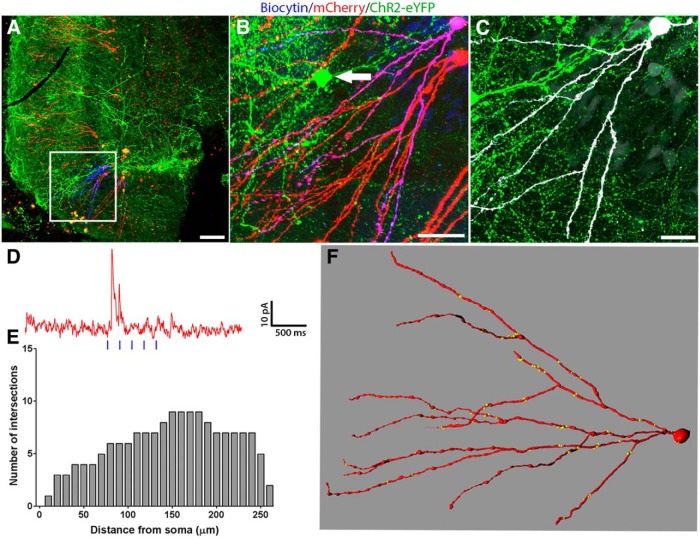
Transplanted GABAergic interneurons innervate adult-born GCs born six weeks post-SE. ***A***, Low-magnification image showing adult-born GCs (red) surrounded by ChR2-eYFP expressing transplants (green) in a mouse that had SE. Boxed region shows the biocytin-filled (blue) adult-born GC that was recorded in this slice using whole-cell patch clamping. ***B***, Magnified view of the boxed region, showing co-labeling with biocytin and mCherry. ***C***, View of the biocytin-filled GC (white) and surrounding axons from transplanted MGE-derived GABAergic interneurons (green). ***D***, Electrophysiological recording from this GC showed a moderate response to optogenetic activation of the ChR2-expressing transplanted interneurons and a distinct IPSC can be seen in response to the first light-pulse. ***E***, Sholl analysis graph of the number of dendritic intersections. ***F***, Complete neuronal reconstruction of this GC and the sites of putative transplant-derived synapses (yellow dots). Scale bars: 100 µm (***A***), 50 µm (***B***), and 20 µm (***C***).

As changes in dendritic morphology are well-documented in hippocampal GCs in TLE, our central question was whether the increased inhibition from the transplanted GABAergic interneurons altered the dendritic growth of adult-born GCs. To address this question, we compared dendritic morphologies in innervated versus non-innervated adult-born GCs, using high-resolution 3D reconstructions and Sholl analyses of 157 RV-labeled adult-born GCs; including 126 GCs from SE mice and 31 GCs from naïve mice. An example of a GC born at six weeks post-SE and innervated by a transplant made at two weeks post-SE is shown in Figure 5. The transplant contained a large VGAT-EYFP^+^ interneuron in the vicinity of this GC (Fig. 5*B*, white arrow), shown at higher magnification in Figure 5*C*. Excitation of the ChR2-expressing axons in this slice evoked IPSCs in host brain GCs (Fig. 5*D*). The Sholl analysis of the dendritic arbor of this GC indicated a relatively normal pattern of dendritic branching (Fig. 5*E*) and the corresponding 3D morphologic reconstruction revealed a high density of transplant-derived putative synapses onto this cell’s dendritic arbor (Fig. 5*F*).

**Figure 6.**
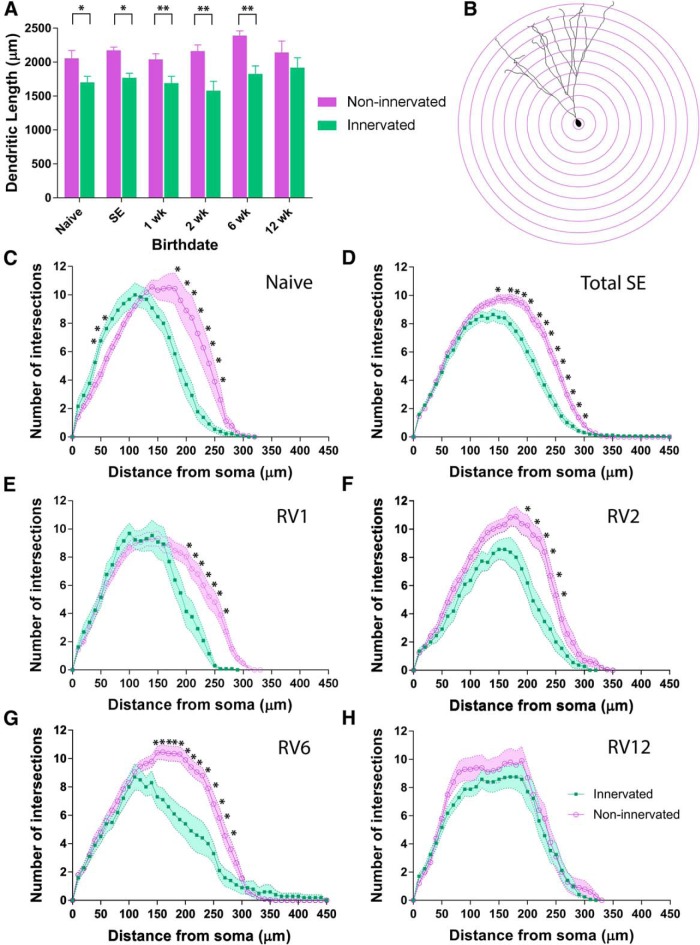
Adult-born GCs innervated by transplanted GABAergic interneurons develop significantly shorter distal dendrites. ***A***, Bar graph of total dendritic lengths in transplant-innervated and non-innervated adult-born GCs in different experimental groups. All neurons were confirmed to be adult-born and RV labeled by immunostaining for mCherry expression. Data for non-innervated GCs is represented by purple bars and innervated GCs are shown in green bars. GCs with high levels of putative synaptic input from the transplanted GABAergic interneurons had significantly shorter dendrites than non-innervated adult-born GCs. Neuronal reconstructions of the GCs in different experimental groups are shown in Extended Data Figures 6-1, 6-2, 6-3, 6-4, 6-5. ***B***, Schematic of the Sholl analysis paradigm for analysis of dendritic branching based on number of dendritic branches intersecting concentric spheres spaced at 10-µm intervals from the soma. ***C–H***, Sholl analyses of dendritic arbors comparing transplant-innervated adult-born GCs (green lines) versus non-innervated GCs (purple lines). Shading represents SEM. ***C***, In the naïve mice, innervated GCs showed reduced dendritic branching both proximally and distally, compared with non-innervated GCs. ***D***, Grouped Sholl data showing significantly reduced dendritic arbors in innervated, adult-born GCs compared to non-innervated adult-born GCs in SE mice with transplants. While the innervated GCs formed similar patterns of proximal branching, they had significantly fewer distal branches, compared to non-innervated adult-born GCs. Significant differences found for radii at 150 μm from the soma (*p* = 0.03) and all radii between 170 and 290 μm from the soma (*p* < 0.01). ***E–H***, Sholl data broken down by the birthdate post-SE of adult-born GCs. Graphs indicate that innervated adult-born GCs had significantly fewer distal dendrites, compared to non-innervated adult-born GCs. ***E***, Innervated GCs born one week post-SE had significantly fewer intersections at radii between 200 and 270 μm from the soma, compared to non-innervated GCs (*p* < 0.05). ***F***, Innervated GCs born two weeks post-SE had significantly reduced dendritic branching at radii 200–250 μm compared to the non-innervated GCs (*p* < 0.05). ***G***, Innervated GCs born six weeks post-SE had significantly fewer dendritic branches at radii 150–270 μm compared to non-innervated GCs (*p* < 0.05). ***H***, Innervated GCs born 12 weeks post-SE showed trend toward reduced branching compared to non-innervated GCs, but this trend did not reach significance. Asterisks indicate statistically significant differences between groups.

To determine whether putative synaptic innervation from the transplanted interneurons induced structural changes to the dendritic arbors of adult-born GCs, we examined the total dendritic lengths (Fig. 6, Table 3). Strikingly, the sizes of the dendritic arbors in GCs with heavy input from the transplanted GABAergic interneurons were significantly smaller, compared to non-innervated GCs in the same animals (total dendritic lengths were on average, 1700.0 ± 92.0 µm in the population of innervated GCs vs an average of 2055.6 ± 116.4 µm in the sample of non-innervated GCs, *p* = 0.0235; Fig. 6*B*). Moreover, a significant effect of transplant-derived innervation was found for GCs born one, two, or six weeks post-SE. When these populations of adult-born neurons were innervated by the transplants, they developed significantly shorter dendrites compared to non-innervated adult-born GCs generated at the same time. Although the RV-labeled GCs born 12 weeks post-SE were also heavily innervated by the transplants and showed a similar trend of having shorter dendrites compared to non-innervated GCs, overall the differences in dendritic arbor size were not statistically significant (1917.0 ± 147 vs 2143.3 ± 167.3 µm, *p* = 0.3211). The results of the statistical tests of significance for all Sholl data are shown in Table 4.

10.1523/ENEURO.0110-18.2019.f6-1 Extended Data Figure 6-1 Neuronal reconstructions of RV-labeled GCs from naïve mice. Representative neuronal reconstructions of the dendritic arbors of adult-born GCs with confirmed expression of RV expression of mCherry. Mature neuronal arbors of innervated and non-innervated GCs are shown approximately eight weeks after RV labeling and nine weeks after MGE transplantation. Scale bar: 100 µm. Download Figure 6-1, TIF file.

10.1523/ENEURO.0110-18.2019.f6-2 Extended Data Figure 6-2 Neuronal reconstructions of GCs labeled with RV one week post-SE. Comparisons of mature dentate GCs that were born one week post-SE in mice that received transplants at two weeks post-SE. Innervated and non-innervated GCs shown eight weeks after MGE transplantation (10 weeks post-SE). Scale bar: 100 µm. Download Figure 6-2, TIF file.

10.1523/ENEURO.0110-18.2019.f6-3 Extended Data Figure 6-3 Neuronal reconstructions of mature GCs labeled with RV two weeks post-SE. Comparisons of mature dentate GCs that were born two weeks post-SE in mice that received transplants at six weeks post-SE. Innervated and non-innervated GCs are shown eight weeks after MGE transplantation (14 weeks post-SE). Scale bar: 100 µm. Download Figure 6-3, TIF file.

10.1523/ENEURO.0110-18.2019.f6-4 Extended Data Figure 6-4 Neuronal reconstructions of eight-week-old GCs labeled with RV six weeks post-SE. Comparisons of mature dentate GC dendritic arbors in cells that were born six weeks post-SE in mice that received MGE transplants two weeks post-SE. Innervated and non-innervated GCs are shown approximately eight weeks after transplantation (14 weeks post-SE). Scale bar: 100 µm. Download Figure 6-4, TIF file.

10.1523/ENEURO.0110-18.2019.f6-5 Extended Data Figure 6-5 Neuronal reconstructions of eight- to 10-week-old GCs labeled with RV 12 weeks post-SE. Representative neuronal reconstructions from populations of innervated and non-innervated GCs that were born 12 weeks post-SE in mice that received transplants at two weeks post-SE. Innervated and non-innervated GCs are shown approximately 18 weeks after transplantation (20 weeks post-SE). Scale bar: 100 µm. Download Figure 6-5, TIF file.

**Table 3.** Total dendritic lengths for different groups of mice

Quantitative measurement	GCs from naïve mice	GCs labeled at 1 week post-SE	GCs labeled at 2 weeks post-SE	GCs labeled at 6 weeks post-SE	GCs labeled at 12 weeks post-SE
Corrected *p* value (using mixed effects model)	0.0235	0.0129	0.0027	0.0010	0.3211
Mean (µm)	Non-innervated: 2055.6 Innervated: 1700.0	Non-innervated: 2039.9 Innervated: 1690.1	Non-innervated: 2161.6 Innervated: 1577.7	Non-innervated: 2390.0 Innervated: 1825.3	Non-innervated: 2143.3 Innervated: 1917.0
Standard deviation	Non-innervated: 493.9 Innervated: 331.8	Non-innervated: 452.5 Innervated: 365.9	Non-innervated: 371.6 Innervated: 466.0	Non-innervated: 316.7 Innervated: 380.1	Non-innervated: 529.1 Innervated: 606.1
*N* (GCs)	Non-innervated: 18 Innervated: 13	Non-innervated: 29 Innervated: 13	Non-innervated: 16 Innervated: 11	Non-innervated: 20 Innervated: 10	Non-innervated: 10 Innervated: 17
*N* (total GCs)	31	42	27	30	27

**Table 4.** Statistical comparisons of Sholl data and significance values for all RV-labeled, adult-born GCs

Radius	Naive	SE	1-week RV	2-week RV	6-week RV	12-week RV
10	0.1727	0.4666	0.8371	0.6229	0.7329	0.5425
20	0.2448	0.4000	0.4370	0.8628	0.9167	0.7634
30	0.2568	0.8828	0.8884	0.3471	0.8933	0.7634
40	0.0634	0.5937	0.9445	0.5976	0.7329	0.7840
50	0.0321	0.3749	0.9445	0.2050	0.7795	0.7634
60	0.0321	0.5723	0.8349	0.3316	0.8529	0.5425
70	0.0321	0.2545	0.7540	0.1376	0.2966	0.5425
80	0.0526	0.4145	0.4370	0.2771	0.4499	0.5425
90	0.0979	0.4885	0.5077	0.3397	0.6101	0.5968
100	0.1765	0.3608	0.4370	0.1376	0.7921	0.6550
110	0.3591	0.2357	0.9445	0.2050	0.7795	0.7328
120	0.9248	0.1969	0.9445	0.1294	0.3682	0.7634
130	0.6053	0.0556	0.9445	0.0489	0.0894	0.7634
140	0.1727	0.1535	0.9445	0.1009	0.1800	0.7634
150	0.1213	0.0314	0.9445	0.1158	0.0039	0.7634
160	0.0953	0.0551	0.9445	0.1158	0.0054	0.7634
170	0.0513	0.0059	0.5871	0.1009	0.0028	0.7634
180	0.0321	0.0026	0.2395	0.0733	0.0028	0.7634
190	0.0321	0.0005	0.0742	0.0660	0.0028	0.7634
200	0.0321	0.0005	0.0174	0.0263	0.0028	0.7634
210	0.0364	0.0005	0.0368	0.0100	0.0028	0.9482
220	0.0321	0.0005	0.0196	0.0090	0.0035	0.7968
230	0.0377	0.0005	0.0196	0.0090	0.0029	0.7634
240	0.0498	0.0005	0.0174	0.0380	0.0056	0.8077
250	0.0526	0.0005	0.0084	0.0270	0.0140	0.9482
260	0.0953	0.0005	0.0084	0.1294	0.0095	0.9482
270	0.1765	0.0005	0.0174	0.1974	0.0307	0.9482
280	0.2245	0.0044	0.0742	0.1974	0.0894	1.000
290	0.3591	0.0071		0.2900	0.1810	0.7634
300	0.7866	0.0559		0.4536	0.4722	0.7634
310		0.2357			0.6300	0.5968
320		0.4962			0.6179	
330		0.9539			0.5304	
340		0.4414			0.3078	
350		0.3117			0.2551	
360		0.2284				
370		0.1969				
380		0.1969				
390		0.1969				
400		0.1969				
410		0.3117				
420		0.3117				
430		0.3117				
440		0.3117				

Statistically significant *p* ≤ 0.05 are shown in red.

Overall, the total dendritic lengths for the innervated versus non-innervated GCs in different groups and the number of GCs that were analyzed for each group were as follows: in naive mice the total dendritic branches were 1700.0 ± 92.0 µm (*n* = 13 transplant-innervated GCs) versus 2055.6 ± 116.4 µm (*n* = 18 non-innervated GCs; *p* = 0.0235). In epileptic mice with RV labeling at one week post-SE, the total dendritic lengths of the transplant innervated versus non-innervated adult-born GCs were 1690.1 ± 101.5 µm (*n* = 13 transplant innervated GCs) versus 2039.9 ± 84.0 µm (*n* = 29 non-innervated GCs; *p* = 0.0129). In epileptic mice with RV labeling at two weeks post-SE, the total dendritic lengths of the transplant innervated versus non-innervated adult-born GCs were 1577.7 ± 140.5 µm (*n* = 11 transplant-innervated GCs) versus 2161.6 ± 93.0 µm (*n* = 16 non-innervated GCs; *p* = 0.0027). In epileptic mice with RV labeling at six weeks post-SE, the total dendritic lengths of the transplant innervated versus non-innervated adult-born GCs were 1825.3 ± 120.2 µm (*n* = 10 transplant innervated GCs) versus 2390.0 ± 70.8 µm (*n* = 20 non-innervated GCs; *p* = 0.0010). In epileptic mice with RV labeling at 12 weeks post-SE, the total dendritic lengths of the transplant innervated versus non-innervated adult-born GCs were 1917.0 ± 147 µm (*n* = 17 transplant innervated GCs) versus 2143.3 ± 167.3 µm (*n* = 10 non-innervated GCs; *p* = 0.3211).

Given that the total dendritic lengths of adult-born GCs were significantly smaller when they were innervated by the MGE transplants, we next compared differences in proximal versus distal dendrite lengths (Fig. 6*C–H*). The GCs from naïve mice that received input from the MGE transplants showed reductions in the lengths of both proximal and distal dendrites, compared with non-innervated GCs (radii 50–70 µm and radii 180–240 µm, *p* < 0.05). However, in epileptic mice, dendritic growth was significantly more stunted distally in GCs that were innervated by the transplants (radii 170–270 µm, *p* < 0.01; Fig. 6*D*), whereas proximally, dendritic growth was unaffected. The most significant differences were found in 3rd through 7th order branches in GCs innervated by the transplants. For example, the average length of 3rd order branches was 64.4 ± 6.8 µm in innervated GCs versus 78.7 ± 4.4 µm in non-innervated GCs (*p* = 0.0390); the average length of 5th order branches in transplant-innervated GCs was 72.2 ± 8.0 versus 108.9 ± 5.1 µm in non-innervated GCs (*p* = 0.0390); the average length of 6th order branches was 34.8 ± 8.6 µm in innervated GCs, versus 71.3 ± 5.5 µm in non-innervated GCs (*p* < 0.0001), and the average length of 7th order branches was 7.5 ± 6.1 µm in innervated GCs versus 22.0 ± 3.9 µm in non-innervated GCs (*p* = 0.0197). We also found that adult-born GCs generated shortly after induction of SE showed the greatest effects; for example, GCs born one week post-SE had significantly shorter 3rd, 4th, and 6th order branches (3rd order branches: innervated 47.6 ± 13.6 µm versus non-innervated 78.9 ± 7.6 µm; *p* = 0.0271); 4th order branches: innervated 73.1 ± 10.7 µm versus non-innervated 95.6 ± 6.0 µm; *p* = 0.0437); 6th order branches: innervated 34.6 ± 15.4 µm versus non-innervated 66.7 ± 8.6 µm; *p* = 0.0438). In contrast, GCs generated at two weeks post-SE only showed significantly shorter 5th order branches (82.5 ± 16.0 vs 117.6 ± 10.2 µm; *p* = 0.0385), and similarly, GCs generated six weeks post-SE only showed significantly shorter 5th order (50.3 ± 17.3 vs 108.1 ± 10.0 µm, *p* = 0.0024) and 6th order branches (24.1 ± 20.5 vs 80.6 ± 11.8 µm; *p* = 0.0102).

We further compared branching patterns based on Sholl analyses for the entire populations of innervated versus non-innervated GCs born one, two, six, and 12 weeks post-SE (Fig. 6*E*–*H*; Table 3). In all of these groups, except for the GCs born 12 weeks post-SE, we found that input from the MGE transplants was linked to a significant reduction in the size of the dendritic arbors, with more restricted growth of the distal dendritic branches. While the GCs generated 12 weeks post-SE did not show statistically significant differences in branching, they did show a similar trend toward shorter distal dendritic branches (Fig. 6*H*; Table 3, dendritic length data).

A larger sample of representative RV-labeled adult-born GCs are shown in Extended Data Figures 6-1, 6-2, 6-3, 6-4, 6-5. While none of the GCs born in naïve mice were dysmorphic, GCs in epileptic mice exhibited highly abnormal arbors with hilar basal dendrites. Taken together, these results indicate that in epileptic and naïve mice, adult-born GCs with putative synapses formed by the MGE transplants had significantly shorter dendritic arbors.

## Discussion

We have used a retroviral approach to label adult-born GCs at successively different times in naïve or epileptic mice in combination with transplantation of MGE progenitors from E13.5 ChR2-EYFP^+^ mouse embryos either before or after RV labeling of adult-born GCs. We allowed for maturation of the transplants and adult-born GCs, then tested for functional integration of the transplants by optogenetically stimulating the transplanted cells, while patch-clamping nearby RV-labeled GCs in hippocampal slices. The GCs were concurrently filled with biocytin during recordings, and after fixing the slices, 3D reconstructions and morphometric analyses were conducted.

The major new findings of this study are that fetal ChR2-expressing mouse GABAergic progenitor transplants in the hippocampus of naïve or epileptic adult mice innervate dentate GCs born weeks or even months after the time of transplantation. Optogenetic stimulation of the transplants induced strong IPSCs in adult-born GCs, and the magnitude of these currents correlated with the number of transplant-derived (putative) GABAergic synapses on these adult-born GCs. Additionally, in either naïve, non-epileptic mice, or epileptic mice, adult-born GCs receiving dense innervation from the transplanted GABAergic interneurons had significantly more compact dendritic arbors, due to reduced growth of distal dendrites.

Remarkably, GCs born during the chronic phase of epilepsy as late as 12 weeks post-SE were heavily innervated by the interneuron transplants and the magnitudes of optogenetically-induced IPSCs were as large as those observed in populations of adult-born GCs generated shortly after transplantation. However, later born GCs showed a more modest trend toward reduced dendritic growth which was not significantly different from non-innervated GCs, suggesting that transplant-induced effects on adult-born GCs and their dendritic growth may wane over time. It was previously shown that astrogliosis increases in the DG during the chronic phase of TLE, leading to compromised GABAergic inhibition, which could reduce efficacy of the both endogenous and transplanted GABAergic interneurons (Eid et al., 2004; Dengler et al., 2017). Indeed, a previous study examining MGE transplants in mice with TLE, showed that seizure suppression did not endure during the later phases of TLE (Henderson et al., 2014). However, further work is needed to determine whether shorter distal branches in adult-born GCs lead to functional changes that reduce either the connectivity or excitability of dentate GCs.

### Relationship to prior studies of GC structural changes in epilepsy

The present study examined structural changes in the dendrites of normotopic adult-born GCs in naïve and SE mice following transplantation and integration of GABAergic progenitors harvested from the fetal mouse MGE. We confined our analyses to normotopic GCs, to increase our sample sizes. It would be important in future studies, however, to investigate whether inputs from transplanted fetal GABAergic cells also restrict the growth of dendrites formed by ectopic GCs born in the epileptic brain, as the ectopic adult-born GCs in particular, are thought to contribute to epileptogenesis and hyperexcitability.

Several studies in rodent models of TLE have compared dendritic arbor complexity in normotopic versus hilar ectopic GCs and reported greater distal GC dendrite branching (Sholl intersections) in epileptic compared to naïve rats (Cameron et al., 2011). Similarly, in tissue from human patients with TLE or extra-hippocampal lesions, GCs were found to have increased dendritic lengths in the inner molecular layer of the DG (von Campe et al., 1997). Our findings suggest that transplant-derived input onto GCs reduces both normal dendritic growth and epilepsy-induced dendritic overgrowth.

### Role of GABA and downstream signaling pathways in structural changes in adult-born GCs

The observed changes in dendrite growth could be mediated by the spontaneous or synaptic release of GABA from the transplanted interneurons. Adult-born GCs receive their first inputs from GABAergic interneurons (Ge et al., 2006). GABA_A_ receptor antagonists have revealed tonic GABA currents in developing GCs as early as 3 d after birth and GABA-mediated synaptic currents as early as 7 d after cell birth (Ge et al., 2006). In contrast, the first glutamatergic currents occur ∼14 d after GC birth (Ge et al., 2006). Newborn GCs express high levels of Na^+^-K^+^-2Cl^−^ transporter NKCC1 and thus, have a high intracellular chloride concentration, which causes GABA to be depolarizing, before a developmental switch to a hyperpolarizing effect of GABA (Ben-Ari et al., 2012). The depolarizing effects of GABA are seen for at least two weeks after GCs are born in the rodent (Ge et al., 2006). The initial GABA-mediated depolarization appears to be important for regulating dendritic growth, as knocking down NKCC1 reduced dendritic length and branching up to 14 d after cell birth (Ge et al., 2006).

Growth-modulating effects of synaptic or extra synaptic GABA could be mediated by a number of different intracellular signaling pathways. For example, a major mechanism for GABA-mediated modulation of intracellular pathways is through activation of voltage-gated calcium channels that are activated by GABA-mediated depolarization, resulting in calcium influx (Schmidt-Hieber et al., 2004). Consistent with this, application of exogenous GABA, to immature cerebellar granule neurons, increased calcium influxes and increased dendritic complexity whereas antagonists of voltage-gated calcium channels, or inhibiting calcium/calmodulin-dependent protein kinase IV, reduced dendritic growth (Redmond et al., 2002; Borodinsky et al., 2003). The effects of GABA and calcium influxes on dendrite growth are likely to be mediated through molecules such as CaMKII and CaMKIV, or downstream signaling proteins, such as mitogen-activated protein kinases (MAPKs) and protein kinase A (PKA; Ghosh and Greenberg, 1995), as inhibition of CaMKIV has been linked to reduced dendritic growth, while increased CaMKIV activation has been shown to increase dendritic growth (Redmond et al., 2002).

Considering the importance of voltage-gated calcium influxes in GC maturation, it will be important to determine whether innervation of adult-born GCs by fetal GABAergic interneurons alters key intracellular calcium-dependent signaling pathways. One downstream effector of CaMKIV is cAMP response element-binding protein (CREB; Redmond et al., 2002). In normal mice, a high percentage of adult-born GCs express phospho-CREB, the active phosphorylated form of CREB (Nakagawa et al., 2002). Retroviral expression of dominant-negative isoforms of phospho-CREB in newly generated GCs reduced dendritic growth and altered the orientation of dendrites (Jagasia et al., 2009). Conversely, upregulating phospho-CREB in adult-born GCs by a pharmacological approach increased dendritic length and branching (Fujioka et al., 2004). Similarly, incubation of primary hippocampal cultures with cAMP agonists increased the expression of phospho-CREB as well as dendritic length and branching (Fujioka et al., 2004). The expression of phospho-CREB in newly-generated GCs begins around 5 d after the cells become postmitotic and lasts up to ∼21 d. The temporal pattern of phospho-CREB expression coincides with the time during which GABA exerts a depolarizing effect on these cells, indicating that GABA-mediated depolarization may play a key role in activation of CREB (Jagasia et al., 2009). One study found that knock-down of NKCC1 in newborn GCs reduced levels of phospho-CREB, indicating a critical role for GABA-mediated depolarization in phosphorylation of CREB at early stages of GC maturation (Jagasia et al., 2009).

Brain-derived neurotrophic factor (BDNF) is a key downstream target of CREB. The expression of phospho-CREB in immature adult-born GCs is correlated with maximal expression of BDNF (Bender et al., 2001). Increased glutamatergic neurotransmission increases BDNF levels, while increased GABAergic inhibition decreases BDNF levels (Zafra et al., 1991). Hippocampal slice cultures treated with BDNF showed increased MAP-2 expression and increased dendritic growth (Marty et al., 1996). Moreover, transplant-derived GABAergic synapses onto adult-born GCs might counteract Hebbian plasticity at excitatory synapses from the entorhinal cortex, as these synapses are also modulated by BDNF (Asztely et al., 2000). Given these prior observations, decreased BDNF release may be responsible for reduced dendritic growth that we observed in adult-born GCs receiving dense inputs from the MGE transplants.

### Summary and conclusions

Our results add to a growing body of literature showing that transplantation of MGE-derived GABAergic interneuron progenitors into the mature brain alters host brain neural circuitry. Here, we provide novel findings showing that putative synaptic input from transplants of GABAergic interneuron progenitors was associated with reduced growth of adult-born GC dendrites. The transplanted interneurons formed putative inhibitory synapses with both proximal and distal dendritic branches, and an overall shortening of dendritic branches was found in adult-born GCs innervated by the transplants in naïve mice. In epileptic mice however, the effect was limited to distal dendrites.

## Synthesis

Reviewing Editor: Alfonso Araque, University of Minnesota Decisions are customarily a result of the Reviewing Editor and the peer reviewers coming together and discussing their recommendations until a consensus is reached. When revisions are invited, a fact-based synthesis statement explaining their decision and outlining what is needed to prepare a revision will be listed below. The following reviewer(s) agreed to reveal their identity: Aaron Taylor, Esther Krook-Magnuson.

The reviewers have found merit and interest on the findings reported. I concur with the reviewers' comments expressing the interest of the study. However, they also expressed some concerns about several issues that need to be addressed and clarified. I also consider pertinent the concerns expressed. We believe that addressing those concerns, which may require less than 2 months' work and will definitely improve the manuscript, is necessary to firmly validate the results.

Specific comments of the reviewers:

Reviewer 1

The authors show that transplantation of GABAergic interneurons reduces the dendritic length and complexity of adult-born granule cells (GCs) within the dentate gyrus. The findings are consistent with related studies (Hsieh & Baraban, 2017; Cunningham, Cho, et al., 2014; Henderson, Gupta, et al., 2014; Peng, Zhang, et al., 2013) and suggest a cellular mechanism for how transplantation of GABAergic interneurons reduces the long-term effects of status epilepticus. Overall, the experiments are rigorously performed and analyzed and are suitable for publication in eNeuro.

Major Concerns:

- The author's definition of GABAergic “synapses” is problematic. In the Methods section, “putative synapses” are defined as puncta “within 0.5 um of the cell that had a diameter of about 0.75 um or larger”. While some of the puncta meeting this definition probably are synaptic contacts, undoubtedly many others are not. For example, in Figure 2E, if one were to draw a 'phantom' dendrite in the same image and apply their definition of a synapse, the phantom would probably be measured as having about the same number of synaptic contacts as the real dendrite. Relatedly, on p.16 the authors claim to be observing GABAergic synapses on “shafts, spines, and spine heads”. While there is some precedent for GABAergic synapses on spines and spine heads, to our knowledge this is a rare occurrence and has not been previously demonstrated on granule cells. (Most GABAergic synapses are on a-spiny regions). Most likely these puncta do not represent synapses. Their quantitative definition is more appropriately described as a measure of the local density of GABAergic axons.

- The correlation between “synapse number” and blue-light triggered IPSC amplitudes is just that. Most likely the transplanted cells have also integrated into the endogenous network of GABAergic cells and so could also act indirectly.

- Given the above concerns, the author's major claim that transplanted “interneurons form functional synapses onto adult-born GCs” is likely but not certain given the data. Only correlative light and electron microscopy would make this claim unequivocal.

Minor Concerns:

-What happened to the arbors of GCs that showed blue-light triggered IPSCs but were not retrovirally labeled (presumably corresponding to pre-existing GCs)?

- The transplant media contained FGF and EGF, both of which have documented effects on dendritic growth. Would the changes to GC arbors be the same if these growth factors were omitted from the transplant media? What would happen to GC arbors near the site of a sham transplantation (i.e. buffer but no cells)?

Reviewer 2

The manuscript reports that granule cells receiving innervation from transplanted inhibitory neurons have shorter dendrites with fewer branches.

The authors show that granule cells targeted by implanted inhibitory neurons have shorter and less branched dendritic arbors compared to granule cells in the same animals not targeted by transplanted interneurons. This is an interesting finding and well supported by the data presented. However, I have the following comments and concerns:

1. Throughout the manuscript it is difficult to discern when cells are or are not putative adult generated cells and when they are RV-confirmed adult generated cells. Similarly, it is difficult to know if granule cells that were not RV-confirmed were granule cells believed to be RV-expressing (but confirmation failed) or if they were intentionally targeted RV-negative cells. The authors should give careful consideration to how they present and analyze the data, and avoid making assumptions about granule cell age without verification, and over-grouping data.

E.g., what supports the claim that granule cells presented in Figure 6 are truly “adult-generated granule cells born after mice develop epilepsy”?

2. Figure 1 shows examples of expression, but no quantification.

3. Figure 2 shows an example recorded cell, but no summary data for RV 1-week post SE + TX 2 weeks post SE animals/cells.

4. Figure 3 shows examples from RV 2 weeks+TX 6 weeks; RV6 weeks+TX2weeks; and TX2weeks+RV12 weeks, but no summary data by injection scheme.

It is also unclear what data is included in Figure 3F. All time points collapsed? Is data from RV 1-week post SE + TX 2 weeks post SE included? If not, why not? Are not-RV-confirmed granule cells included? Where any differences in innervation noted between RV and not-RV granule cells?

5. It is unclear how or when the animals implanted with intracranial electrodes (p. 11), or the vEEG data collected, were used in the research, if at all.

6. It is unclear if the transplant targeted neurons are receiving more total inhibitory input, which would have direct implications on the interpretations the authors are making. The authors presumably have sIPSC data for the GCs included, which could be analyzed to address this issue.

7. The statement “The effect of GABAergic innervation .... After transplantation” in the Significance Statement and similar sentences throughout should be careful not to over interpret the lack of statistical significance in the 12wk group, given the trend seen.

8. Statements in the introduction and discussion are often overly simplified, and “but see” comments would improve these sections (this includes correlations between seizure burden and ectopic GCs, and the long-term effects of transplants regarding seizure suppression).

9. The manuscript appears to be currently internally inconsistent. P17 “8 were confirmed to have RV-laeling and 5/8 showed light-induced IPSCs”. “In total, ... and all showed strong light-induced inhibitory currents”. I do not see how 5/8 can be all. The total of 14 RV confirmed GCs also does not seem to match the total of the individual numbers given.

Minor:

1. Data presented in Figure 3-1 does not address the question it is slated to answer (“What functional subtypes of interneurons in the transplant innervate adult-born GCs?”).

2. Information in the methods section should include survival rate, if any animals not displaying SE were included in either group, what the 'naïve' group entails, and if “electrophysiology” refers to slice electrophysiology (and/or video-EEG monitoring).

3. The authors might consider rearranging the manuscript for flow. For example, Figure 3-1 seems poorly placed.

4. The process for determining cell number in the cell suspension (p.8, in the context of “Transplantation of MGE-derived GABAergic progenitors”) should be described.

5. The temperature of the ACSF in the recording chamber (p.9) should be included.

6. The method for seizure detection (p.11) lacks detail and should be expanded (or excluded if not being used).

7. In “Phenotypic Characterization of Transplanted Cells” the process for counting single-labeled and double-labeled MGE-derived cells is not described. Were all cells counted in every section?

8. A rationale for the exclusion of female mice should be provided.

9. Under “Statistical Analyses” please include a brief description of how your data will be presented throughout (e.g. mean +/- st.dev.) and ensure that all data points listed in the text are listed in that format. Additionally, throughout the text, specify which statistical test is used for each reported p-value.

10. Define all acronyms, and use the same acronym consistently (e.g. SOM vs SST)

11. Figure 2: The addition of arrows in panels A, B, and C denoting which neuron is filled in panel D would be helpful. Additionally, it appears as though the eYFP signal in the center of the image in panel C has been photobleached, perhaps giving a false perception of the density of ChR2-eYFP in the region (especially given the density of the eYFP signal in panel D).

12. p.20 of the text states that there is an interneuron with varicose dendrites denoted by an arrow in Figure 5B -- this is not apparent in the figure itself. Similarly, Figure 5E and 5F are not correctly cited in the text.

13. Figure 6 legend would benefit from inclusion of more information. What does the “SE” bar include? What do the shadings in C-H represent?

14. Figures 6-2 to 6-5: in addition to time post SE, please include also the time since transplantation.

15. Table 3: specify the comparison (i.e. comparing innervated versus non-innervated GCs), the statistical test used, and if p-value reported are corrected or uncorrected.

16. p. 23 “To identify GCs, we used a retroviral approach to label adult-born GCs at successively different times after SE induction and the development of TLE.” -- The development of TLE was not shown.

17. Bottom of p. 23, top of p. 24: “the number of inhibitory synaptic inputs...” “...that received dense innervation from...” This phrasing should be updated to clarify that these are putative inhibitory synaptic inputs.
