# Supplementary figures and images for: Restrained Dendritic Growth of Adult-Born Granule Cells Innervated by Transplanted Fetal GABAergic Interneurons in Mice with Temporal Lobe Epilepsy
Source: eNeuro. 2019 Apr 9;6(2):ENEURO.0110-18.2019. doi: 10.1523/ENEURO.0110-18.2019 (PMC6497906; doi:10.1523/ENEURO.0110-18.2019)

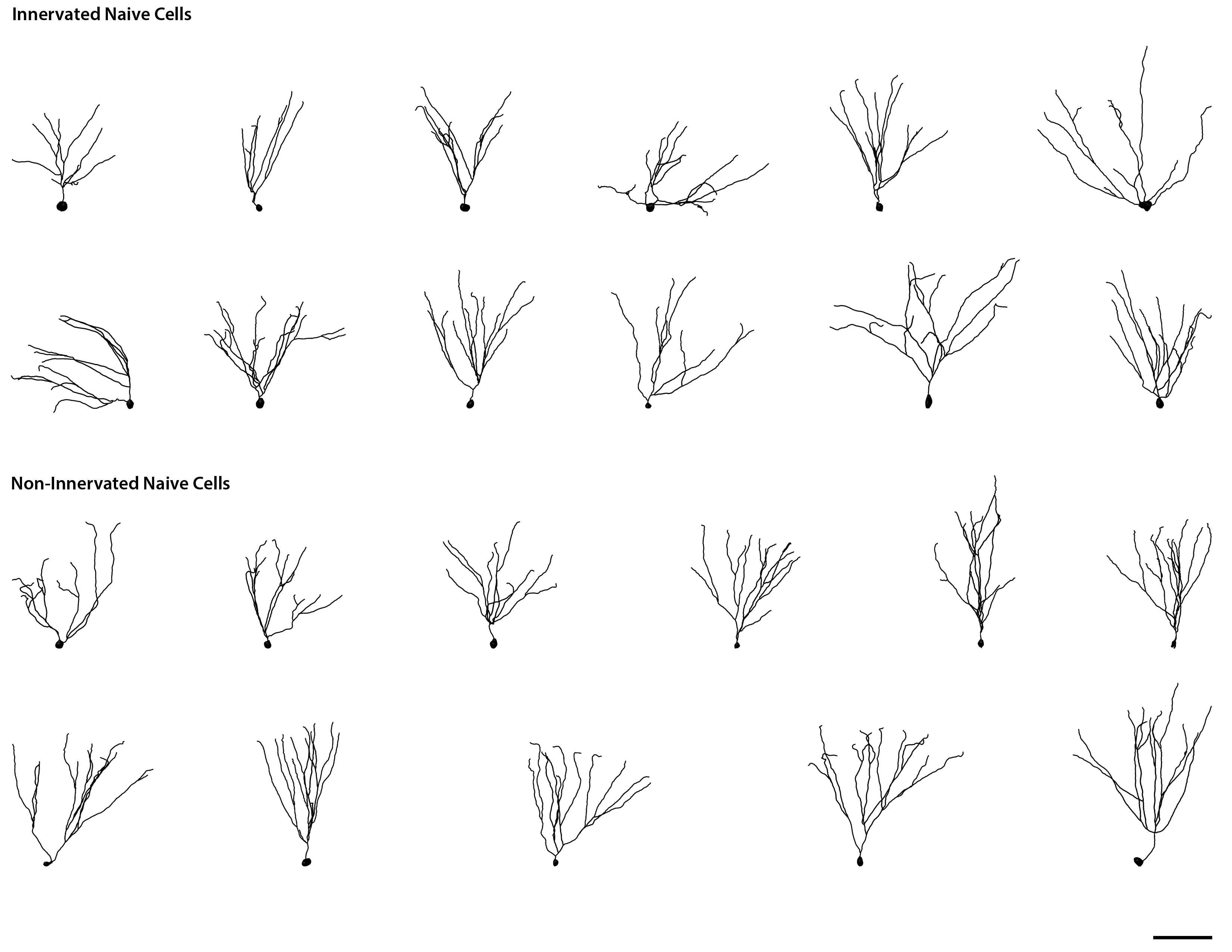

Supplement: Extended Data Figure 6-1 — Neuronal reconstructions of RV-labeled GCs from naïve mice. Representative neuronal reconstructions of the dendritic arbors of adult-born GCs with confirmed expression of RV expression of mCherry. Mature neuronal arbors of innervated and non-innervated GCs are shown approximately eight weeks after RV labeling and nine weeks after MGE transplantation. Scale bar: 100 µm. Download Figure 6-1, TIF file. [file enu002192905so2.tif]

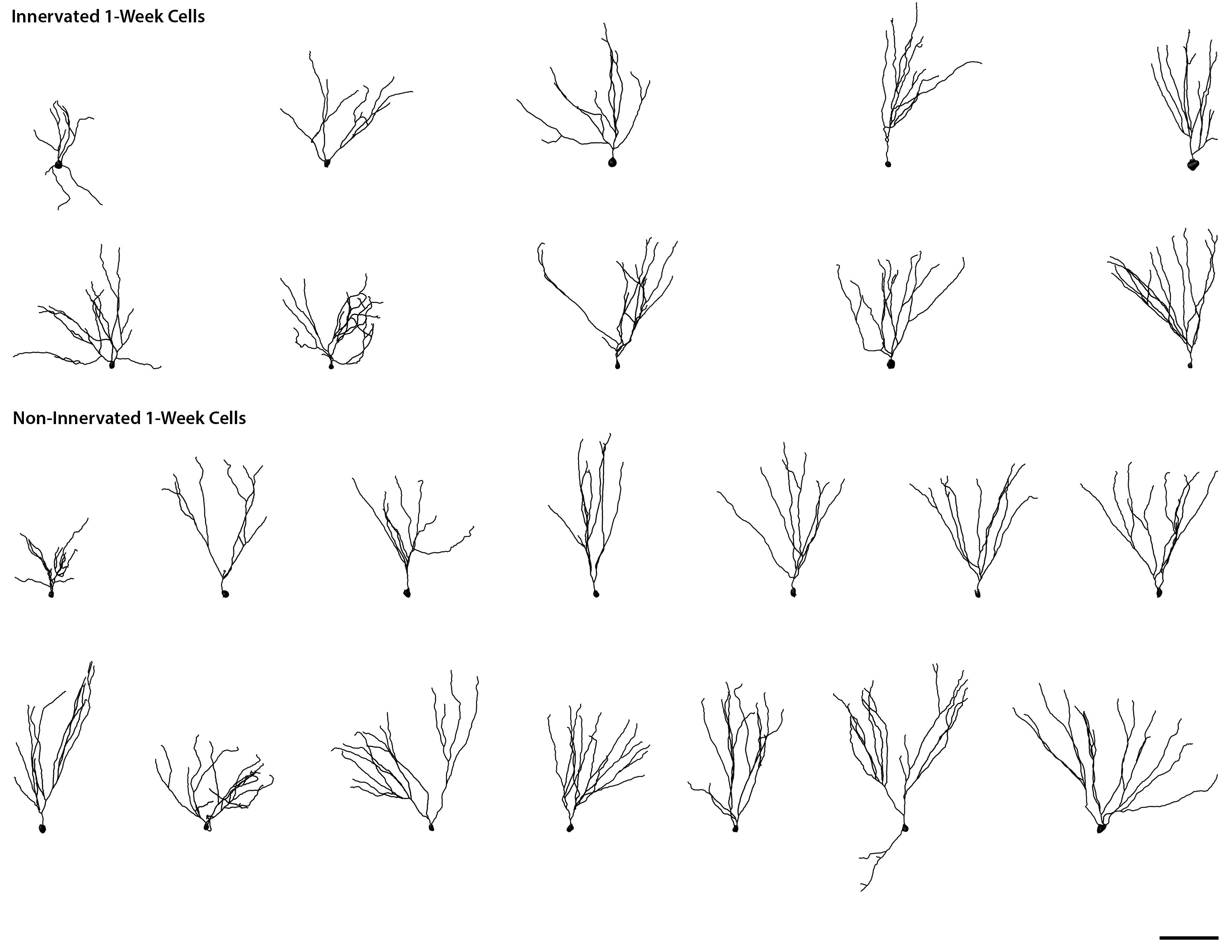

Supplement: Extended Data Figure 6-2 — Neuronal reconstructions of GCs labeled with RV one week post-SE. Comparisons of mature dentate GCs that were born one week post-SE in mice that received transplants at two weeks post-SE. Innervated and non-innervated GCs shown eight weeks after MGE transplantation (10 weeks post-SE). Scale bar: 100 µm. Download Figure 6-2, TIF file. [file enu002192905so3.tif]

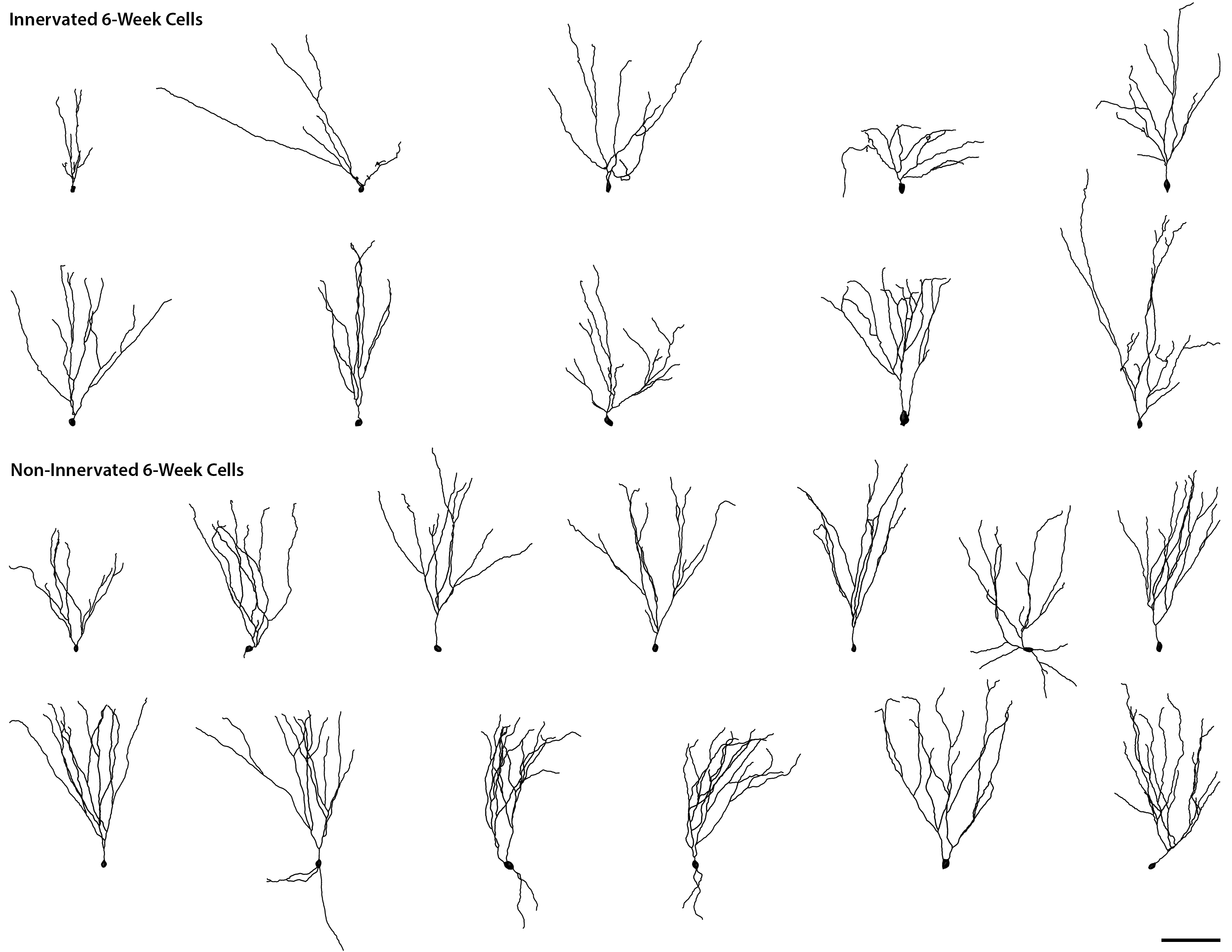

Supplement: Extended Data Figure 6-4 — Neuronal reconstructions of eight-week-old GCs labeled with RV six weeks post-SE. Comparisons of mature dentate GC dendritic arbors in cells that were born six weeks post-SE in mice that received MGE transplants two weeks post-SE. Innervated and non-innervated GCs are shown approximately eight weeks after transplantation (14 weeks post-SE). Scale bar: 100 µm. Download Figure 6-4, TIF file. [file enu002192905so5.tif]

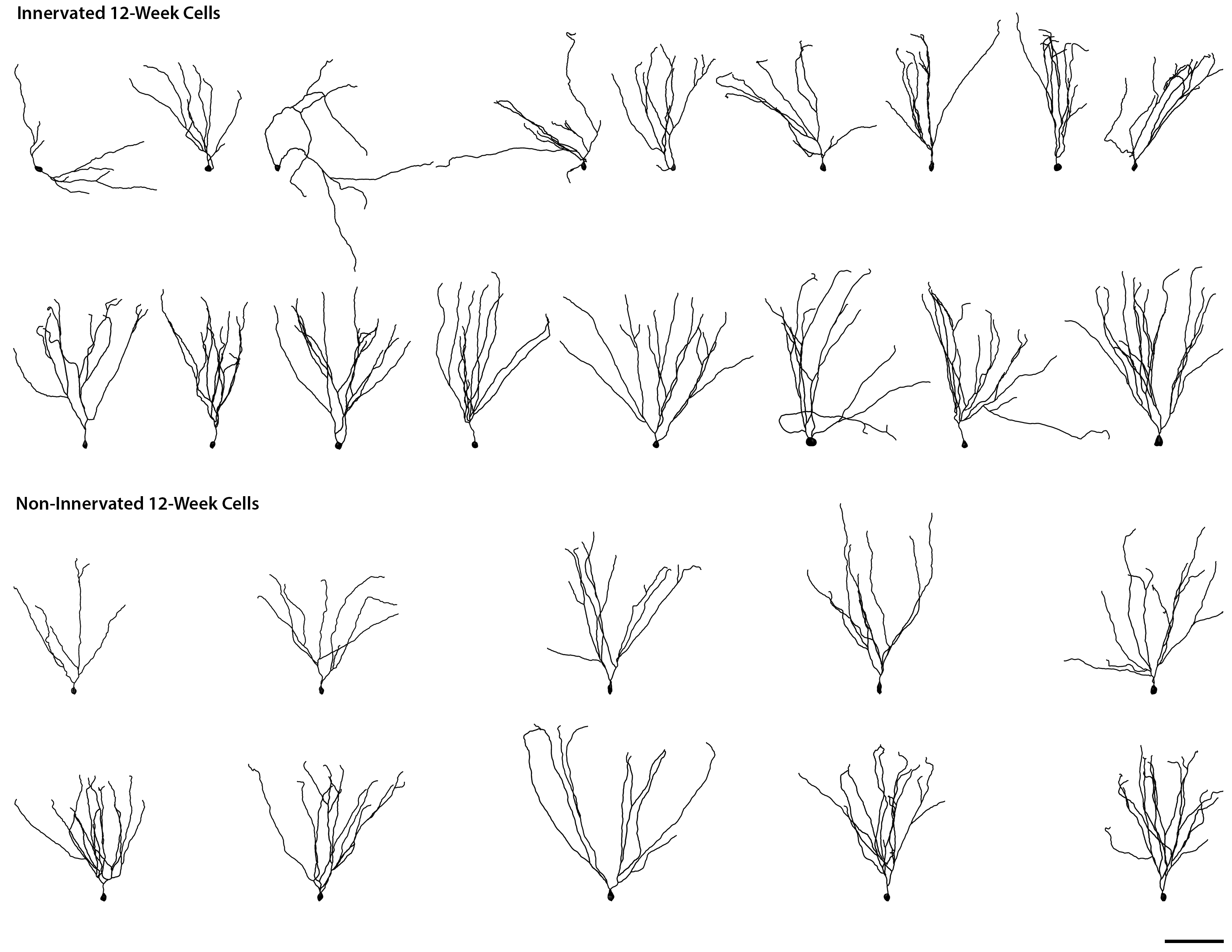

Supplement: Extended Data Figure 6-5 — Neuronal reconstructions of eight- to 10-week-old GCs labeled with RV 12 weeks post-SE. Representative neuronal reconstructions from populations of innervated and non-innervated GCs that were born 12 weeks post-SE in mice that received transplants at two weeks post-SE. Innervated and non-innervated GCs are shown approximately 18 weeks after transplantation (20 weeks post-SE). Scale bar: 100 µm. Download Figure 6-5, TIF file. [file enu002192905so6.tif]
